# CD8^+^ T cells recognizing a neuron-restricted antigen injure axons in a model of multiple sclerosis

**DOI:** 10.1172/JCI162788

**Published:** 2023-11-01

**Authors:** Benjamin D.S. Clarkson, Ethan M. Grund, Miranda M. Standiford, Kanish Mirchia, Maria S. Westphal, Liz S. Muschler, Charles L. Howe

**Affiliations:** 1 Department of Neurology,; 2 Department of Laboratory Medicine and Pathology,; 3 Center for Multiple Sclerosis and Autoimmune Neurology,; 4 Mayo Graduate School,; 5 Medical Scientist Training Program,; 6 Division of Experimental Neurology, Mayo Clinic, Rochester, Minnesota, USA.

**Keywords:** Immunology, Neuroscience, Multiple sclerosis, Neurological disorders, T cells

## Abstract

CD8^+^ T cells outnumber CD4^+^ cells in multiple sclerosis (MS) lesions associated with disease progression, but the pathogenic role and antigenic targets of these clonally expanded effectors are unknown. Based on evidence that demyelination is necessary but not sufficient for disease progression in MS, we previously hypothesized that CNS-infiltrating CD8^+^ T cells specific for neuronal antigens directly drive the axonal and neuronal injury that leads to cumulative neurologic disability in patients with MS. We now show that demyelination induced expression of MHC class I on neurons and axons and resulted in presentation of a neuron-specific neoantigen (synapsin promoter–driven chicken ovalbumin) to antigen-specific CD8^+^ T cells (anti-ovalbumin OT-I TCR-transgenic T cells). These neuroantigen-specific effectors surveilled the CNS in the absence of demyelination but were not retained. However, upon induction of demyelination via cuprizone intoxication, neuroantigen-specific CD8^+^ T cells proliferated, accumulated in the CNS, and damaged neoantigen-expressing neurons and axons. We further report elevated neuronal expression of MHC class I and β2-microglobulin transcripts and protein in gray matter and white matter tracts in tissue from patients with MS. These findings support a pathogenic role for autoreactive anti-axonal and anti-neuronal CD8^+^ T cells in MS progression.

## Graphical abstract

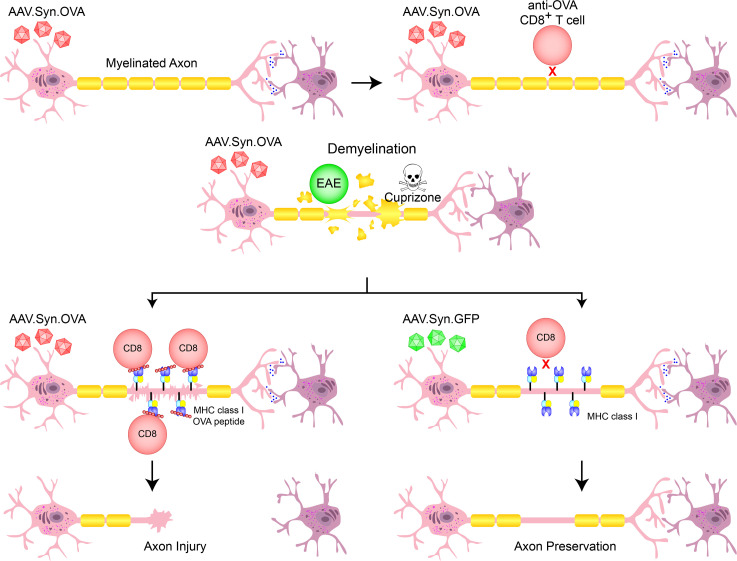

## Introduction

Multiple sclerosis (MS) (1, 2) affects nearly 3 million people worldwide, with an estimated annual economic burden in excess of $100B USD. Progressive disease accounts for the bulk of this burden (3). Acute loss of neurologic function associated with relapse in MS is largely dependent on axon conduction defects triggered by focal demyelination and inflammation (4–6). Resolution of symptoms during remission results in remyelination and compensatory changes in axonal ion channel distribution/function (7–9). However, in many patients, axonal loss and functional disability accumulate over time, either secondary to a relapsing-remitting course or as a primary progressive disease evolution (10–12). Inflammatory demyelination is the predominant pathological hallmark of MS and current therapies for MS were approved based on the ability to prevent clinical relapses and reduce the number of inflammatory demyelinated lesions detected by MRI. However, these therapies do not prevent disease progression (13–15). There is, therefore, a critical unmet need to identify the pathogenic mechanisms driving MS progression and to develop therapies that limit or reverse such progressive disease. Gray matter lesions, brain atrophy, and neuronal and axonal loss, rather than clinical relapses or demyelinated lesion load, best correlate to long-term patient outcomes (10, 16, 17). Histopathological analysis of biopsy and autopsy material from MS patients reveals diffuse axonal and neuronal loss in both normal-appearing white and gray matter. While the specific pathological processes that underlie axonal loss or initiate gray matter lesions in MS remain unknown (18), current evidence suggests that cortical and deep gray matter lesions are more likely to occur in brain regions abundantly connected via axonal tracts to regions with existing lesions (19, 20). This suggests that axons are the key locus of injury diffusion and progression in MS.

MHC class I–restricted CD8^+^ T cells predominate in active MS lesions, outnumbering CD4^+^ T cells by 10-fold or more (21). In addition, CD8^+^ T cells, but not CD4^+^ T cells, are clonally expanded in the brain, CSF, and blood of MS patients, suggesting recognition of specific antigenic targets (22, 23). Critically, the number of CD8^+^ T cells within MS lesions correlates with active axonal injury (24) and axonal injury correlates with disability (25, 26). We have previously shown that demyelination is necessary but not sufficient for axonal injury and functional deficits in a mouse model of MS, while CD8^+^ T cells are both necessary, and within the context of demyelination, sufficient to elicit profound axonal injury and loss of function (27–30). MHC class I expression is observed in developing neurons, where it plays a central role in synaptic pruning (31–35). In contrast, adult neurons do not express MHC class I molecules under homeostatic conditions (36). However, adult neurons upregulate MHC class I in response to a number of pathogenic and inflammatory stimuli, including blockade of spontaneous electrical activity (37, 38) and in response to IFN treatment (36, 39, 40). Neurons also upregulate MHC class I during infection and neuroinflammation, leading to neuronal presentation of self and foreign antigens to CD8^+^ T cells (36, 40, 41). We have previously demonstrated that axon-specific stimulation with IFN-γ drives upregulation of molecules involved in antigen processing and presentation on MHC class I in cultured mouse (41) and human (40) neurons and axons. These findings suggest that class I–restricted cytotoxic CD8^+^ T cells directed against axonal and neuronal self-antigens may be key mediators of the injury that results in irreversible loss of function in MS patients (42). We now provide evidence that demyelination drives presentation of a neuron-specific neoantigen on axonal MHC class I and that neuroantigen-specific CD8^+^ T cells infiltrate the demyelinated CNS and injure antigen-presenting axons.

## Results

### Neuronal expression of antigen processing and presentation genes is induced by inflammation and demyelination.

**Figure 1:**
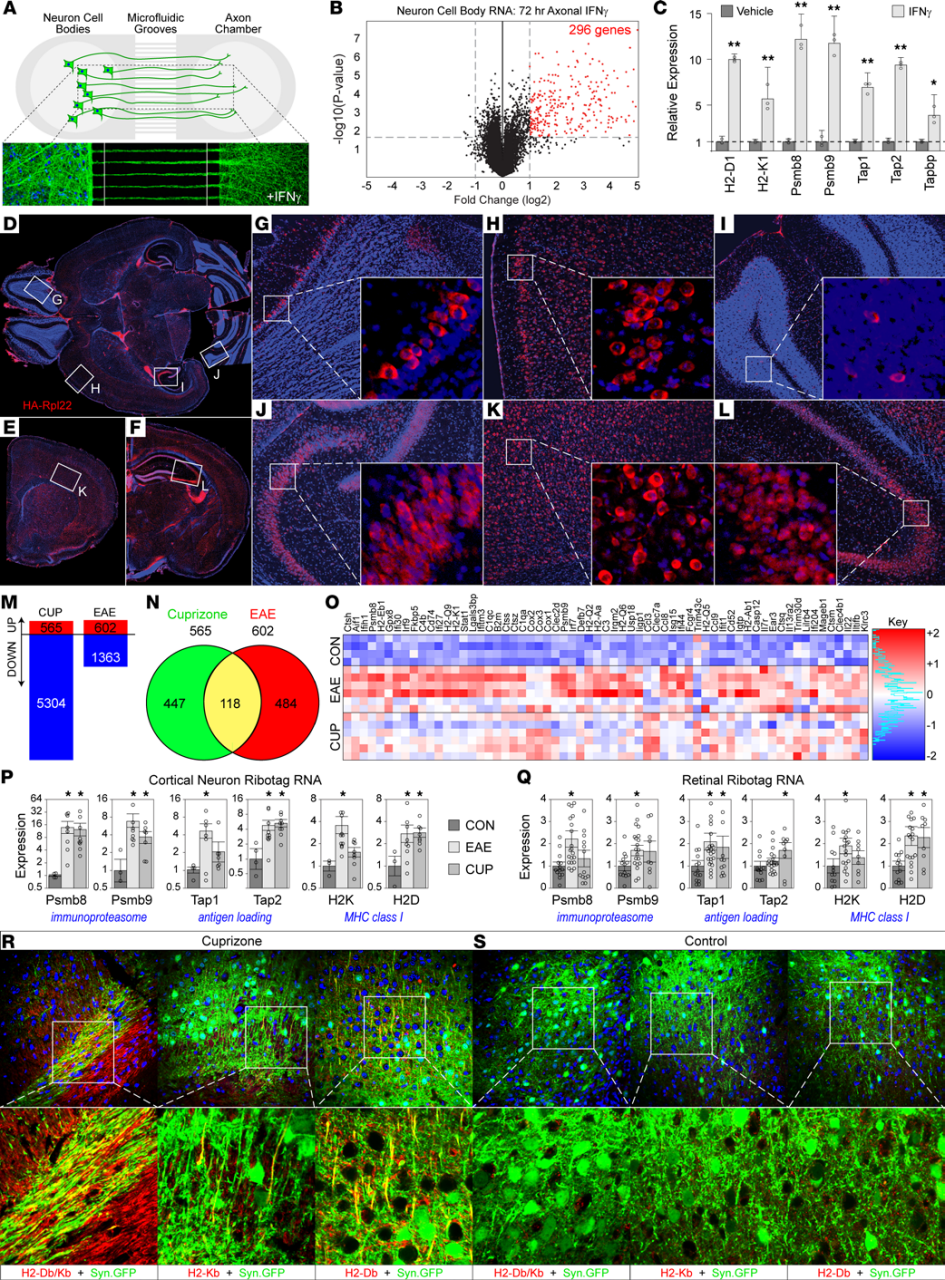
Antigen processing and presentation genes are upregulated in neurons during CNS demyelination. (**A**) Schematic of the microfluidic device used to physically isolate pure axonal fields from neuron cell bodies. Representative fluorescence microscopy image of AAV.Syn.GFP-transduced cortical neurons projecting long axons in such a device. For inflammatory stimulation, IFN-γ was added to the distal axon chamber. (**B**) Axonal fields of mouse cortical neurons were stimulated with 100 ng/mL IFN-γ or vehicle for 72 hours. RNA was isolated from the cell body chamber and processed by microarray analysis of gene transcripts; 296 genes were identified as significantly upregulated (*P* < 0.05, fold change> 2; genes listed in Supplemental Figure 1). (**C**) RT-PCR analysis confirmed that multiple antigen processing and presentation genes were retrogradely upregulated in neurons by axonal IFN-γ stimulation (*n* = 3 samples per condition, **B** and **C**). Photomicrographs of axial (**D**, **G**, **H**, **I**, **J**) and coronal (**E**, **F**, **K**, **L**) sections from Syn.Cre × RPL22 mice stained with DAPI (blue; nuclei) and for anti-hemagglutinin (red; HA-tagged ribosomal subunit of Rpl22) in neurons of cortex, hippocampus, and cerebellum. Boxes indicate high-magnification insets (i–vi). Representative of *n* = 5 mice. Original magnification, ×5 (**D**–**F**) and ×10 (digitally magnified insets in **G**–**L**). (**M**) Differentially expressed genes (DEGs) among mRNAs isolated specifically from neurons in Syn.Cre × RPL22 mouse cerebral cortices 18 days after induction of MOG EAE or after 6 weeks on cuprizone diet (CUP), compared with controls. (**N**) Venn diagram of DEGs upregulated in **M**. (**O**) Heatmap showing levels of selected transcripts undergoing active translation in neurons. (**P** and **Q**) Relative expression of MHC class I and associated antigen presentation genes in neurons isolated as above (**P**) or from retina (**Q**) at 18 days of EAE or after 6 weeks on cuprizone diet (CUP), compared with controls. Each dot represents an individual animal. (**R** and **S**) Representative photomicrographs (×40 magnification) of MHC class I expression (red; H2-D^b^ and/or H2-K^b^) in cortical neurons and axons (green; AAV.Syn.GFP) counterstained with DAPI (blue; nuclei) in cuprizone (**R**) and control mice (**S**). Representative of *n* > 5 mice per condition. Error bars are the 95% CI. *P < 0.05; **P < 0.01 by multiple unpaired *t* tests with Welch’s correction and Benjamini-Hochberg correction for multiple comparisons (**C**) or Kruskal-Wallis 1-way ANOVA with Dunn’s pairwise comparison (**P** and **Q**).

To identify transcriptional patterns induced in neurons by retrograde inflammatory signaling, we cultured primary mouse cortical neurons in axon-isolating microfluidic chambers (Figure 1A) (40). Pure axonal fields were stimulated for 72 hours with IFN-γ (100 ng/mL) and RNA was isolated from the cell body chamber. Microarray analysis identified 296 genes that were significantly upregulated (*P* < 0.05, fold change > 2) (Figure 1B). Many of these genes clustered into related gene families or signaling pathways, including the complement pathway, inflammatory chemokines, MHC class I and antigen presentation genes, and IFN response genes (Supplemental Table 1 and Supplemental Figure 1; supplemental material available online with this article; https://doi.org/10.1172/JCI162788DS1). RT-PCR analysis confirmed that axonal IFN-γ stimulation induced robust retrograde neuronal upregulation of genes involved in antigen processing and presentation (Figure 1C).

RiboTag mice carry a targeted mutation of the ribosomal protein L22 (Rpl22) locus harboring a *loxP*-flanked WT exon 4 followed by an identical exon that is terminally tagged with 3 copies of hemagglutinin (HA). We crossed RiboTag mice with mice expressing Cre recombinase under control of the synapsin promoter (Syn.Cre) to facilitate the isolation of polyribosome-associated RNA transcripts specifically from neurons. To verify the neuronal specificity and the distribution of RiboTag expression across the neuraxis in these mice (Syn.Cre × Rpl22), we labeled axial (Figure 1, D and G–J) and coronal brain sections (Figure 1, E, F, K, and L) with anti-HA antibody. Extensive labeling of neurons was noted in the cortex and hippocampus and was confirmed by colocalization with MAP2 (Supplemental Figure 2). To examine the impact of inflammatory demyelination on the neuronal transcriptome, we induced experimental autoimmune encephalomyelitis (EAE) with a myelin oligodendrocyte glycoprotein peptide (MOG_35–55_) in Syn.Cre × Rpl22 mice and extracted neuronal RNA from the cortex at the peak of disease (18 days after induction). We also fed Syn.Cre × Rpl22 mice cuprizone to induce demyelination and collected cortical RNA after 6 weeks on chow. Retinal RNA was collected from mice under the same conditions. Microarray analysis (Figure 1, M–O) identified 565 genes upregulated in cortical neurons in mice demyelinated by cuprizone (*n* = 6) and 602 genes upregulated in cortical neurons in mice with EAE (*n* = 6), relative to controls (*n* = 4). Gene ontology terms overrepresented in the EAE and cuprizone groups are shown in Supplemental Table 2. A total of 118 genes were shared between these 2 distinct demyelinating insults (Figure 1N; heatmap of shared genes is shown in Figure 1O). These analyses revealed that demyelination, whether it is induced by inflammation or toxin, drives transcriptional programs in neurons that are enriched for antigen processing and presentation genes. RT-PCR validation confirmed robust upregulation of multiple MHC class I antigen processing and presentation genes in cortical neurons in both EAE and cuprizone mice (Figure 1P). This finding suggests the existence of a common mechanism of demyelination-induced neuronal responses that converge on MHC class I antigen presentation. Furthermore, the same transcriptional pattern was observed in the retina (Figure 1Q) in these 2 models. Given the unique polarity of nerve fibers in the optic nerve and the absence of myelin within the retina, this finding provides in vivo evidence that MHC class I genes are retrogradely induced by neuron-intrinsic signals triggered by distal axonal demyelination. Finally, mice were inoculated by intracranial injection of an adeno-associated virus (AAV) encoding GFP under the control of the synapsin promoter (AAV.Syn.GFP), resulting in strong expression of GFP within cortical projection axons (Figure 1, R and S). These animals were fed cuprizone or control chow for 6 weeks and then brain sections were labeled with an antibody against the MHC class I molecules H2-D^b^ and H2-K^b^. Cuprizone (Figure 1R), but not control, mice (Figure 1S and Supplemental Figure 3) exhibited pronounced MHC class I expression on GFP^+^ axons. This observation indicates that demyelination not only induces antigen processing and presenting transcriptional programs in neurons, but also results in expression of MHC class I protein on axons.

### Axons present self-antigen on MHC class I in response to inflammation.

**Figure 2:**
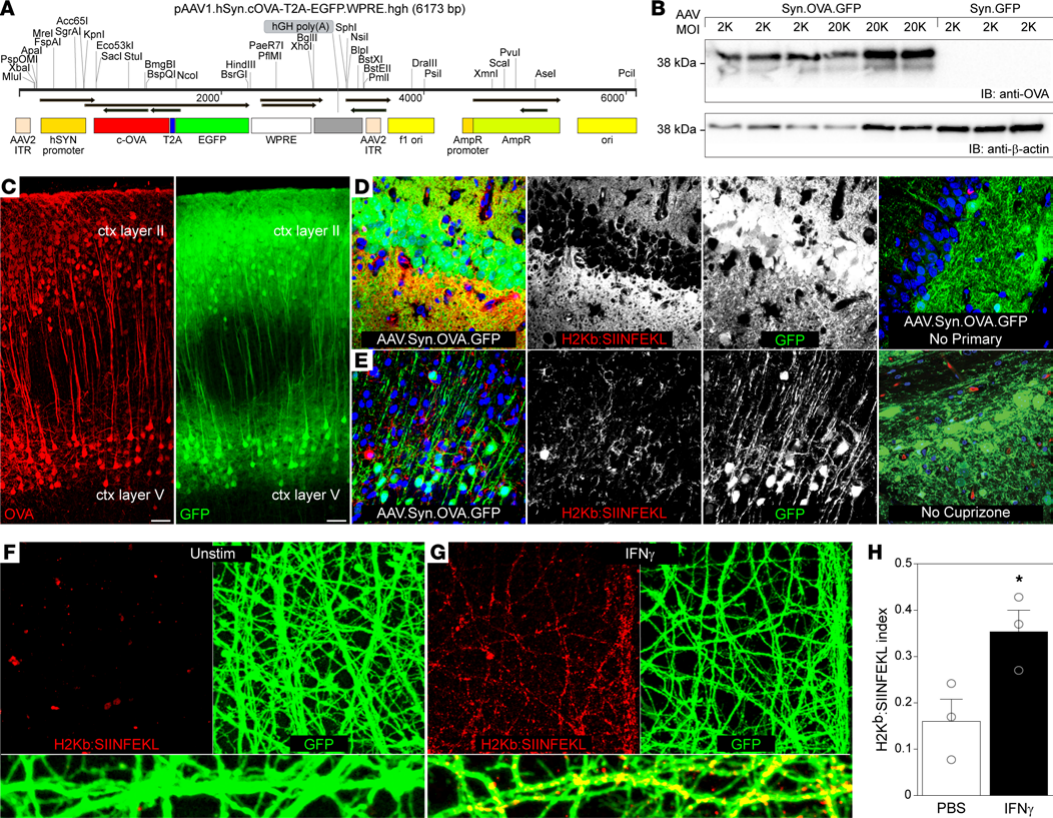
Axons present self-antigen on MHC class I in response to inflammation. (**A**) Schematic showing elements of the adeno-associated virus (AAV) plasmid encoding synapsin (Syn) promoter–driven expression of cytosolic ovalbumin (OVA) with an axon-targeting motif coupled to EGFP by a T2A autocatalytic sequence (AAV.Syn.OVA.GFP). (**B**) Representative Western blot of lysates prepared from DIV 12 cortical neurons transduced at plating with 2,000 (2K) or 20,000 (20K) MOI of AAV.Syn.OVA.GFP or AAV.Syn.GFP, probed with anti-OVA. (**C**) Representative (*n* = 5 mice) photomicrographs of AAV.Syn.OVA.GFP-transduced mouse cortex showing immunostaining for OVA (red) in GFP-expressing neurons (green). Scale bars: 50 μm. (**D** and **E**) Representative images of the OVA peptide SIINFEKL presented on the H2-K^b^ MHC class I molecule (anti-H2K^b^:SIINFEKL; red) colocalized with GFP^+^ neurons and axons in mice demyelinated by cuprizone (×60 magnification). The absence of staining with no primary antibody and in non–cuprizone-treated AAV.Syn.OVA-GFP–transduced mice is shown on the right. (**F** and **G**) H2K^b^:SIINFEKL (red) on OVA-GFP^+^ axons (green) from cortical neurons cultured in microfluidic chambers following axonal stimulation with PBS (**F**) or 100 ng/mL IFN-γ for 72 hours (**G**). Yellow puncta in the higher-magnification panels at the bottom indicate expression of SIINFEKL peptide–loaded MHC class I on axons (×40 magnification). (**H**) Quantification of H2K^b^:SIINFEKL labeling on axons in **F** and **G** relative to GFP. In vitro data are representative of at least 3 independent experiments. Error bars are SEM. *P < 0.01 by unpaired, 2-tailed *t* test (**H**).

To investigate the functional capacity of neurons to present antigen to cognate CD8^+^ T cells, we engineered an AAV that drives neuron-restricted expression of the prototypical neoantigen ovalbumin (OVA) downstream of the synapsin promoter (Figure 2A). In this construct, full-length cytosolic OVA is preceded by an axon-targeting motif derived from the Kv3 potassium channel and is expressed in frame with enhanced GFP (EGFP) separated by a T2A autocatalytic cleavage site (AAV.Syn.OVA.GFP). Transduction of primary cortical neurons with AAV.Syn.OVA.GFP resulted in OVA expression, as detected by Western blotting (Figure 2B; see complete unedited blots in the supplemental material). Furthermore, stereotactic intracerebral injection of the vector resulted in robust expression of OVA and GFP in cortical neurons (Figure 2C). Cuprizone-induced demyelination of AAV.Syn.OVA.GFP-transduced mice led to expression of H2-K^b^ molecules loaded with the OVA-derived SIINFEKL peptide along GFP-positive axons and terminals in the hippocampus (Figure 2D) and cortex (Figure 2E). No H2-K^b^:SIINFEKL staining was observed on GFP^+^ axons in the absence of demyelination in AAV.Syn.OVA.GFP-transduced mice (Figure 2E). Likewise, no H2-K^b^:SIINFEKL staining was present on GFP^+^ axons in cuprizone-demyelinated mice transduced with AAV.Syn.GFP (Supplemental Figure 4). Cortical neurons cultured in microfluidic isolation chambers were also transduced with AAV.Syn.OVA.GFP and axons were subsequently stimulated with IFN-γ. Unstimulated axons showed strong GFP fluorescence but did not express H2-K^b^ loaded with the OVA-derived SIINFEKL peptide (Figure 2F). Likewise, neurons transduced with control AAV.Syn.GFP did not exhibit H2-K^b^:SIINFEKL immunostaining in the presence or absence of IFN-γ stimulation (Supplemental Figure 5). In contrast, IFN-γ stimulation induced a dense distribution of H2-K^b^:SIINFEKL–positive puncta along GFP-positive axons, indicating intraneuronal antigen processing and MHC class I peptide loading induced by inflammation (Figure 2, G and H).

### Peripheral immune surveillance of a neuron-restricted antigen is increased during CNS demyelination.

**Figure 3:**
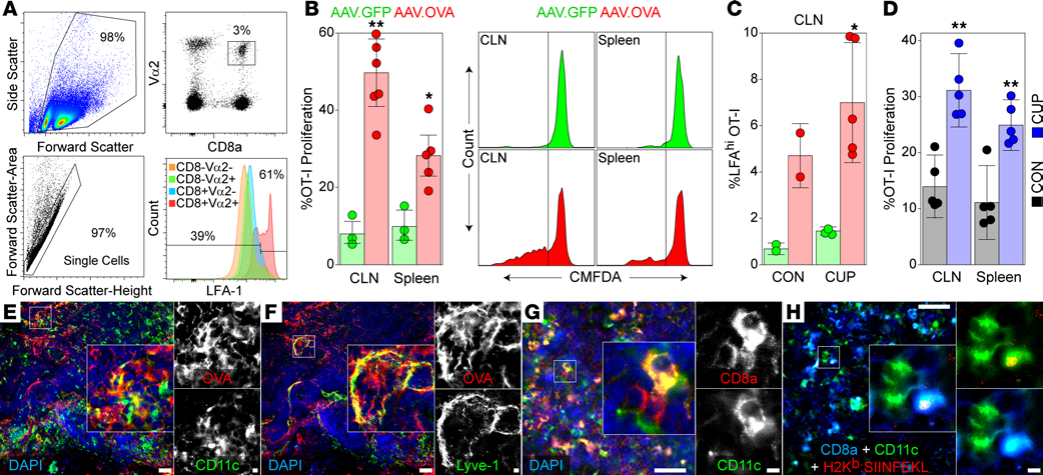
Immune surveillance of a neuron-restricted antigen is increased during CNS demyelination. (**A**) Gating strategy for identifying CD8^+^Vα2^+^ OT-I T cells among deep cervical lymph node (CLN) cells in mice receiving adoptive transfer of 2 × 10^6^ CMFDA-labeled OT-I cells 5 days prior to analysis. (**B**) OT-I T cell proliferation in deep CLNs and spleen of cuprizone-fed mice transduced with AAV.Syn.GFP (AAV.GFP, green) or AAV.Syn.OVA.GFP (AAV.OVA, red). (**C**) Percentage of CLN OT-I T cells exhibiting an LFA-1^hi^ phenotype in mice transduced with AAV.Syn.GFP (green) or AAV.Syn.OVA.GFP (red) and fed either control chow (CON) or cuprizone (CUP). (**D**) Proliferation of adoptively transferred OT-I T cells in deep CLNs or spleen of mice transduced with AAV.Syn.OVA.GFP and fed control chow (gray) or cuprizone (blue). (**E** and **F**) Representative images of brain-draining deep CLNs showing CD11c^+^ dendritic cells (**E**; green) and Lyve-1^+^ stromal cells (**F**; green) colocalized with OVA (red) in cuprizone-fed mice transduced by intracranial inoculation with AAV.Syn.OVA.GFP. The same field from tissue labeled with 4 colors has been false colored to present CD11c and Lyve-1 individually. (**G**) Image of deep CLN showing CD11c^+^ (green) and CD8a^+^ (red) dendritic cells. (**H**) Representative image showing CD11c^+^ (green) and CD8a^+^ (blue) cells in the deep CLN of cuprizone-fed mice transduced by intracranial inoculation with AAV.Syn.OVA.GFP present SIINFEKL on H2-K^b^ (red). Images representative of 5 mice. Long scale bars: 100 μm; short scale bars: 10 μm. Error bars show the 95% CI. *P < 0.05; **P < 0.01 by 1-way ANOVA with Benjamini-Hochberg correction for multiple comparisons (**C**) or multiple unpaired *t* tests with Holm-Šidák multiple comparison correction (**D**).

Immune surveillance of CNS-restricted antigens remains incompletely understood (44, 45). Recently, immune surveillance of oligodendrocyte-restricted (46) and forebrain-restricted (47) neoantigens was described. We sought to determine whether antigens restricted to neurons — which represent a postmitotic cell type with low turnover and scant expression of MHC class I in the mature, healthy CNS — are similarly sampled by the peripheral immune system. Mice were transduced by intracranial inoculation with AAV.Syn.OVA.GFP or AAV.Syn.GFP and then fed cuprizone or control chow for 6 weeks. OT-I T cells (2 × 10^6^ cells per mouse) labeled with 5-chloromethylfluorescein diacetate (CMFDA) were adoptively transferred into these animals 5 days prior to collection of deep cervical lymph nodes (CLNs) and spleen. Recovered CD8a^+^ cells were gated on Vα2 and Vβ5.1 expression to isolate OT-I T cells (Figure 3A). Demyelinated mice expressing neuron-specific OVA exhibited increased proliferation of antigen-specific T cells in both the deep CLNs and spleen relative to animals transduced with GFP only (Figure 3B). Moreover, LFA-1 expression, indicative of T cell activation, was increased on OT-I T cells recovered from mice transduced with AAV.Syn.OVA.GFP, relative to mice transduced with AAV.Syn.GFP, and this activation was enhanced in mice with cuprizone-induced demyelination (Figure 3C). Among the other activation markers that were investigated, CD44 expression was also upregulated on adoptively transferred OT-I.Thy1.1 CD8^+^ T cells in recipients transduced with OVA relative to mice transduced with GFP only (Supplemental Figure 6). Likewise, OT-I T cell proliferation in CLNs and spleen was increased in the cuprizone-treated mice (Figure 3D), suggesting that demyelination led to increased local sampling of neuronal antigens and/or increased drainage of neuronal antigens to peripheral lymphoid organs. This is supported by evidence of OVA within CD11c^+^ dendritic cells (Figure 3E) and Lyve-1^+^ stromal cells (Figure 3F) in the deep CLNs of demyelinated mice. We also observed CD11c^+^CD8a^+^ dendritic cells (Figure 3G) that were positive for SIINFEKL-loaded H2-K^b^ (Figure 3H) in AAV.Syn.OVA.GFP-transduced cuprizone-demyelinated mice, indicating uptake and presentation of neuron-derived peptides within peripheral lymphoid organs. No SIINFEKL-loaded H2-K^b^ was detected in the deep CLNs in demyelinated mice transduced with AAV.Syn.GFP (Supplemental Figure 7). These findings suggest ongoing immune surveillance and sampling of neuron-restricted antigens in the healthy CNS, with increased activation and proliferation of neuronal antigen–specific CD8^+^ T cells induced by demyelination.

### CD8^+^ neuronal antigen–specific T cells are recruited to the demyelinated CNS.

**Figure 4:**
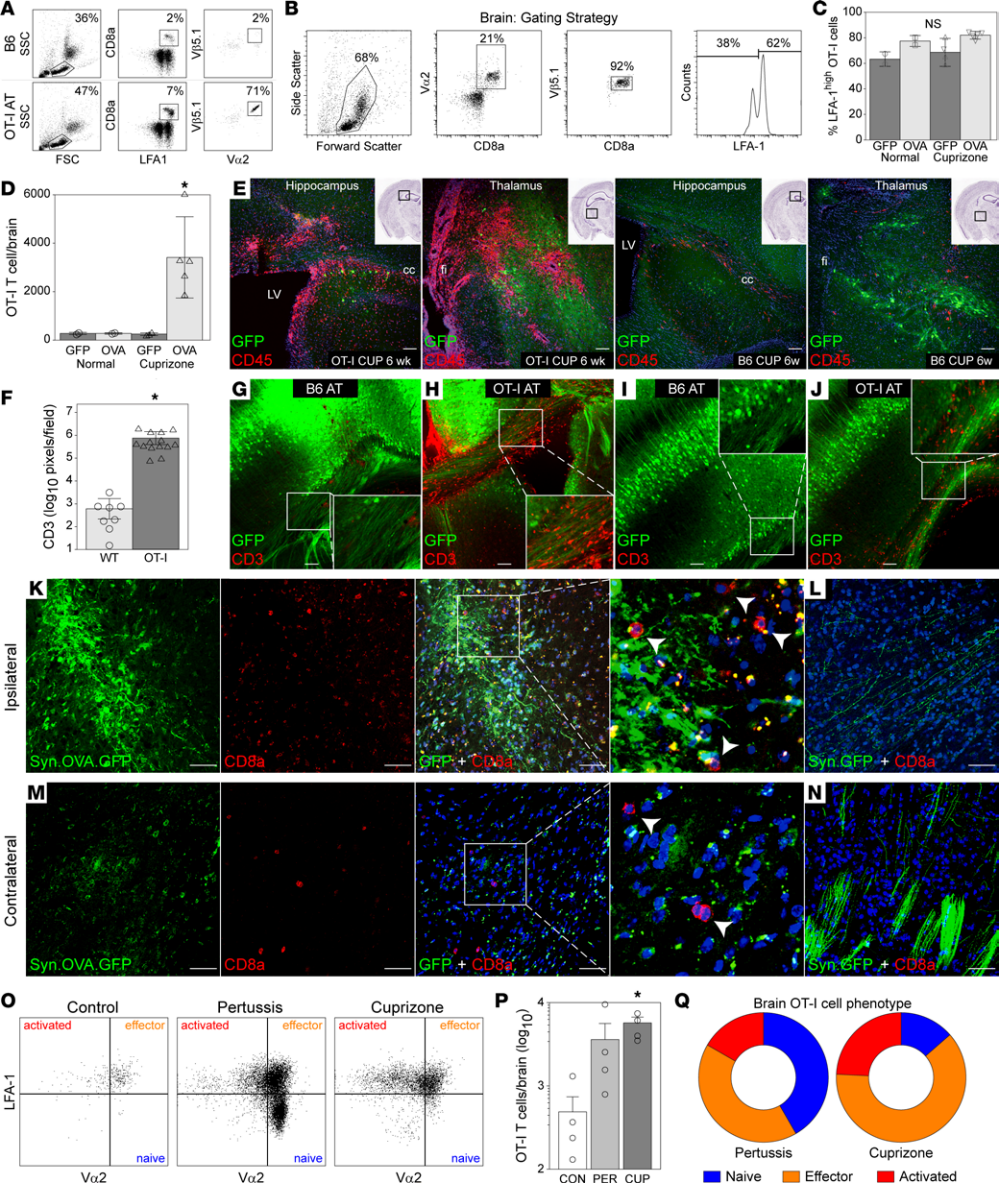
CD8^+^ neuronal antigen–specific T (nasT) cells are recruited to the demyelinated CNS. (**A**) Representative flow plots of blood cells stained as indicated for OT-I markers in lightly irradiated mice adoptively transferred with B6 or OT-I CD8^+^ T cells. (**B**) Gating strategy for identifying CD8^+^Vα2^+^Vβ5.1^+^ OT-I cells among brain-infiltrating lymphocytes in cuprizone-fed mice transduced with AAV.Syn.OVA.GFP. (**C**) Percentage of brain-infiltrating OT-I CD8^+^ T cells expressing high levels of the LFA-1 activation marker in mice transduced with either AAV.Syn.GFP (GFP) or AAV.Syn.OVA.GFP (OVA) and fed either normal chow or cuprizone for 6 weeks. (**D**) Total number of OT-I T cells accumulating in the brain in mice transduced with either AAV.Syn.GFP (GFP) or AAV.Syn.OVA.GFP (OVA) and fed either normal chow or cuprizone for 6 weeks. (**E**) Representative images of CD45^+^ (red) cells in proximity to GFP^+^ neurons and axons (green) in the hippocampus and thalamus of AAV.Syn.OVA.GFP-transduced mice adoptively transferred with OT-I or B6 T cells after 6 weeks on cuprizone. Insets show H&E-stained sections, with boxes indicating location of the fluorescent image. (**F**) Quantitation of CD3^+^ cells in the brain following adoptive transfer of B6 (WT) or OT-I T cells into AAV.Syn.OVA.GFP-transduced mice fed cuprizone for 6 weeks. (**G**–**J**) Representative images of CD3^+^ T cells (red) in the brain in cuprizone-fed mice transduced with AAV.Syn.OVA.GFP (**G** and **H**) or AAV.Syn.GFP (**I** and **J**) receiving adoptive transfer (AT) of B6 (**G** and **I**) or OT-I (**H** and **J**) T cells. (**K** and **L**) Representative images of CD8^+^ T cells (red) adjacent to GFP^+^ cells and axons on the ipsilateral side of brain transduced with AAV.Syn.OVA.GFP (**K**) or AAV.Syn.GFP (**L**) in mice fed cuprizone for 6 weeks prior to adoptive transfer of OT-I T cells. (**M** and **N**) CD8^+^ T cells (red) adjacent to GFP^+^ structures in the contralateral cortex under the same conditions as (**K**) and (**L**). Images are representative of *n* = 5 AAV.Syn.OVA.GFP mice and *n* = 2 AAV.Syn.GFP mice. (**O**) Flow plots showing LFA-1^+^ brain-infiltrating OT-I T cells in mice receiving intraperitoneal pertussis toxin (200 ng at 96 and 48 hours prior to tissue collection) relative to mice fed cuprizone for 6 weeks. (**P**) Quantitation of brain accumulation of OT-I T cells from mice in **O**. (**Q**) Pie chart representation of the percentage of brain-infiltrating OT-I T cells exhibiting a naive (LFA-1^lo^Vα2^hi^; blue), effector (LFA-1^hi^Vα2^hi^; orange), or activated effector (LFA-1^hi^Vα2^lo^; red) phenotype in **O**. Scale bars: 100 μm. Error bars show the 95% CI; each symbol represents 1 animal. *P < 0.01 by 1-way ANOVA with Benjamini-Hochberg correction (**C** and **D**), unpaired, 2-tailed *t* test (**F**), or Kruskal-Wallis 1-way ANOVA with Dunn’s pairwise comparison (**P**).

Irradiation (4 Gy) of B6 host mice 4 hours prior to adoptive transfer of 2 × 10^6^ CD8^+^ T cells from OT-I donors led to reconstitution of approximately 70% of the circulating CD8^+^ T cell pool with Vβ5.1^+^ OT-I T cells (Figure 4A). Light irradiation created an environment that was permissive to OT-I expansion (Supplemental Figure 8) without exerting any effect directly on CNS infiltration (Supplemental Figure 9). Exploiting this strategy, B6 mice were intracranially transduced with AAV.Syn.OVA.GFP or AAV.Syn.GFP, fed cuprizone or control chow for 6 weeks, and then reconstituted with OT-I effectors. Brain-infiltrating lymphocytes were gated on Vα2 and Vβ5.1 expression to isolate OT-I T cells (Figure 4B). While the percentage of brain-infiltrating OT-I T cells expressing the LFA-1 activation marker did not differ between conditions (Figure 4C), the total number of OT-I T cells in the brain was robustly increased in demyelinated mice transduced with AAV.Syn.OVA.GFP (Figure 4D), indicating that both demyelination and the expression of cognate antigen were required for the accumulation of antigen-specific effectors in the brain. This effect was reflected in the presence of CD45^bright^ cells proximal to GFP^+^ axons and neurons in the hippocampus and thalamus of demyelinated mice transduced with AAV.Syn.OVA.GFP (Figure 4E). We also observed an increase in CD3^+^ cells (Figure 4F) associated with GFP^+^ axons in the corpus callosum and cortex of demyelinated mice transduced with AAV.Syn.OVA.GFP receiving adoptive transfer of OT-I T cells (Figure 4, H and J), but not in the same mice receiving B6-derived T cells (Figure 4, G and I). These OT-I T cells primarily accumulated adjacent to GFP^+^ cortical and hippocampal neurons with projections into the corpus callosum, adjacent to GFP^+^ axons within the corpus callosum, and in periventricular areas near GFP^+^ neurons and axons. Further analysis of demyelinated mice transduced with AAV.Syn.OVA.GFP (Figure 4, K and M) or AAV.Syn.GFP (Figure 4, L and N) following adoptive transfer of OT-I T cells revealed the presence of CD8a^+^ T cells associated with GFP^+^ axons and cells in the ipsilateral cortex (Figure 4, K and L) and with GFP^+^ axons in the contralateral (Figure 4, M and N) cortex only in mice expressing OVA in neurons.

To determine whether the accumulation of CD8^+^ T cells in the brain was due to specific retention of antigen-specific cells or was the result of nonspecific demyelination-induced effects on T cell access to the CNS, we compared the activation profile of brain-infiltrating CD8^+^ OT-I T cells in mice transduced with AAV.Syn.OVA.GFP that were either treated with cuprizone for 6 weeks or treated with pertussis toxin (200 ng/mouse, i.p.) at 96 and 48 hours prior to tissue collection. OT-I T cells were found in the brain at high levels in either treatment condition (Figure 4, O and P). However, the cells in the demyelinated brain were predominantly effector T cells and highly activated effector cells exhibiting downregulation of the T cell receptor (TCR) α chain (Figure 4, P and Q), while many of the cells in mice treated only with pertussis were antigen-naive LFA-1^lo^ T cells (Figure 4, O and Q). These findings suggest that CNS recruitment of effector CD8^+^ T cells directed against a neuron-restricted antigen is a selective process that requires drivers associated with demyelination rather than just nonselective permeability of the blood-brain barrier.

### Brain-infiltrating neuronal antigen–specific T cells secrete proinflammatory cytokines and display an activated phenotype.

**Figure 5:**
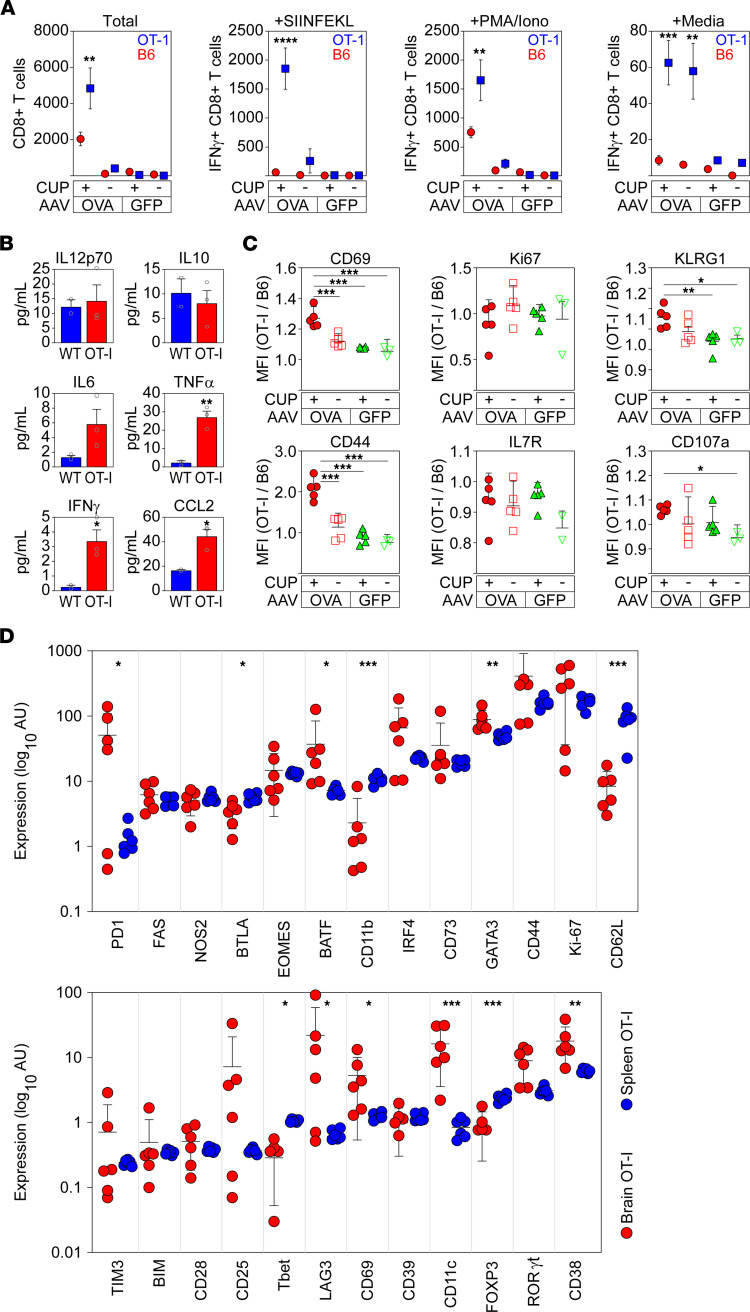
Brain-infiltrating nasT cells exhibit an activated phenotype. (**A**) Number of adoptively transferred B6 (CD8^+^Vα2^–^Thy1.1^+^) and OT-I (CD8^+^Vα2^+^Thy1.1^+^) T cells recovered from the brain 5 days after reconstitution with a 1:1 mixture into Thy1.1-negative hosts transduced with AAV.Syn.OVA.GFP or AAV.Syn.GFP and fed control chow (–) or cuprizone (+) for 6 weeks. *n* = 3–5 mice per condition. Quantitation of IFN-γ production in response to ex vivo stimulation of these brain-infiltrating CD8^+^ T cells with SIINFEKL peptide, PMA + ionomycin, or vehicle. (**B**) Cytokine production by brain-infiltrating CD8^+^ T cells after 24 hours in culture. CD8^+^ T cells were magnetically purified from brain-infiltrating leukocytes collected from AAV.Syn.OVA.GFP-transduced, cuprizone-fed B6 mice that received adoptive transfer of B6 (WT) or OT-I T cells. (**C**) Levels of surface activation markers on brain-infiltrating CD8^+^ T cells collected from mice treated as in **A**, shown as the ratio of antigen-specific T cell MFI (CD8^+^Vα2^+^Thy1.1^+^) to antigen-naive T cell MFI (CD8^+^Vα2^–^Thy1.1^+^). (**D**) Analysis of expression level of 25 markers in brain-infiltrating OT-I T cells (red) vs. splenic OT-I T cells (blue) using mass cytometry. Mice were transduced with AAV.Syn.OVA.GFP and demyelinated with cuprizone for 6 weeks prior to adoptive transfer of the OT-I T cells. Error bars are the 95% CI. *P < 0.05; **P < 0.01; ***P < 0.001; ****P < 0.0001 by 2-way ANOVA with Holm-Šidák multiple-comparison test (**A**), unpaired, 2-tailed *t* test (**B**), 1-way ANOVA with Dunnett’s multiple-comparison test (**C**), or multiple unpaired *t* tests with Benjamini-Hochberg correction for multiple comparisons (**D**).

B6 mice (Thy1.1-negative) were transduced by intracranial inoculation with AAV.Syn.OVA.GFP or AAV.Syn.GFP, fed cuprizone or control chow for 6 weeks, irradiated, and then adoptively transferred with a 1:1 mixture of OT-I.Thy1.1^+^ and B6.Thy1.1^+^ T cells. Analysis of antigen-specific versus nonspecific adoptively transferred cells revealed that OT-I cells were preferentially recruited into the brain at high numbers in cuprizone-demyelinated AAV.Syn.OVA.GFP-transduced mice (Figure 5A) compared with nondemyelinated mice. Furthermore, following ex vivo stimulation of isolated brain-infiltrating CD8^+^ T cells for 5 hours with SIINFEKL peptide (10 μg/mL), OT-I cells (CD8^+^Vα2^+^Thy1.1^+^) produced IFN-γ (Figure 5A), indicating that these brain-infiltrating cells are functional effector T cells. Adoptively transferred brain-infiltrating B6-derived cells (CD8^+^Vα2^–^Thy1.1^+^) did not respond to SIINFEKL stimulation. Polyclonal ex vivo stimulation with phorbol myristic acid (PMA) (50 ng/mL) and ionomycin (1 μM) induced IFN-γ production in a fraction of both OT-I (CD8^+^Vα2^+^Thy1.1^+^) and B6 (CD8^+^Vα2^–^Thy1.1^+^) brain-infiltrating T cells (Figure 5A), indicating the presence of effector CD8^+^ T cells among both brain-infiltrating adoptively transferred Thy1.1 populations. In contrast with brain-infiltrating T cells from AAV.Syn.GFP-transduced mice, a small number of brain-infiltrating OT-I cells from demyelinated AAV.Syn.OVA.GFP-transduced mice exhibited spontaneous IFN-γ production without further stimulation. This production may indicate a response induced by endogenously presented OVA peptide just prior to cell harvest. Of interest, a small number of brain-infiltrating OT-I cells isolated from nondemyelinated AAV.Syn.OVA.GFP-transduced mice also produced IFN-γ in response to peptide antigen or PMA/ionomycin stimulation. Similarly, a small number of these OT-I cells also produced IFN-γ in the unstimulated condition, in contrast with cotransferred B6 T cells. Brain-infiltrating OT-I T cells also secreted higher levels of IL-6, TNF-α, IFN-γ, and CCL2 following ex vivo incubation for 24 hours relative to cotransferred B6 cells. Secretion of IL-12p70 and IL-10 was not different between OT-I and B6 brain-infiltrating CD8^+^ T cells (Figure 5B). Furthermore, relative to co-infiltrating nonspecific CD8^+^ T cells, OT-I T cells isolated from the brain of AAV.Syn.OVA.GFP-transduced, cuprizone-demyelinated mice exhibited increased surface expression of CD69, CD44, KLRG1, and CD107a, with no difference observed in expression of Ki67 or IL-7R (Figure 5C). Brain-infiltrating OT-I T cells also differed from splenic OT-I T cells in adoptively transferred mice. B6 mice were transduced by intracranial inoculation with AAV.Syn.OVA.GFP, fed cuprizone for 6 weeks, irradiated, and then adoptively transferred with OT-I.Thy1.1^+^ T cells. At 5 days after reconstitution, Thy1.1^+^ cells were isolated from brain and spleen and analyzed by mass cytometry to determine expression levels of 25 markers. Brain-infiltrating antigen-specific CD8^+^ T cells exhibited higher expression of PD-1, BATF, GATA3, LAG3, CD11c, and CD38, with a trend toward increased expression of IRF4, CD44, Ki67, CD25, and RORγt. Brain-infiltrating cells exhibited decreased expression of BTLA, CD11b, CD62L, T-bet, and FOXP3 (Figure 5D).

### CD8^+^ neuronal antigen–specific T cells injure axons and neurons in the demyelinated brain.

**Figure 6:**
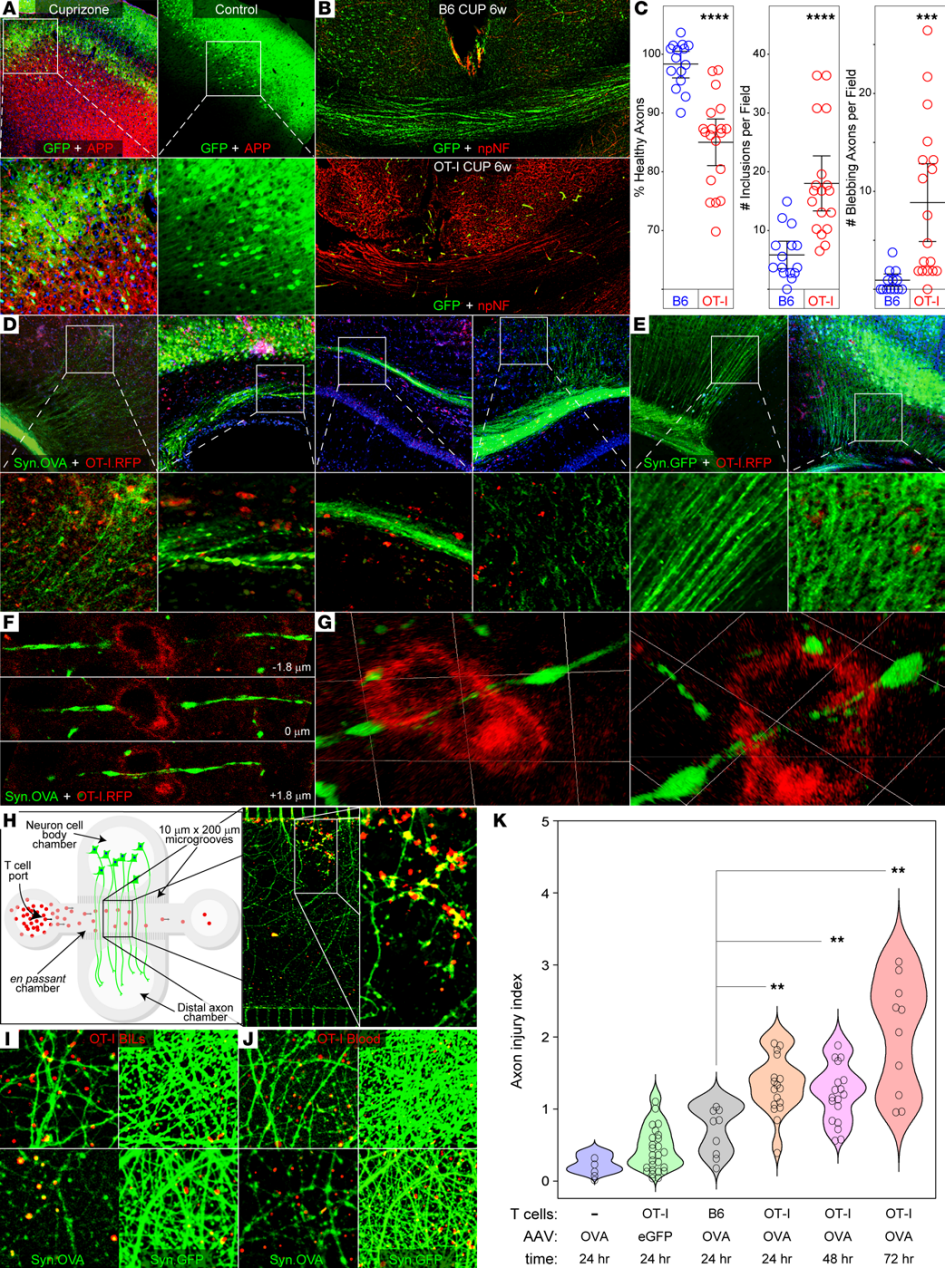
CD8^+^ nasT cells injure neurons in the demyelinated brain. (**A**) Representative images showing APP (red) in cortex 5 days after adoptive transfer of OT-I T cells into AAV.Syn.OVA.GFP-transduced mice fed cuprizone or control chow for 6 weeks. Boxes indicate higher magnification images shown directly beneath each panel. (**B**) Representative images showing nonphosphorylated neurofilament (npNF; red) and GFP^+^ axons (green) in the corpus callosum in AAV.Syn.OVA.GFP-transduced B6 or OT-I mice fed cuprizone for 6 weeks. (**C**) Quantitation of axonal injury parameters following adoptive transfer of B6 (blue) or OT-I (red) T cells into AAV.Syn.OVA.GFP-transduced mice fed cuprizone for 6 weeks. (**D**) Representative images of RFP^+^ OT-I T cells (red) in proximity to GFP^+^ axons (green) in the cortex and corpus callosum 5 days after adoptive transfer into AAV.Syn.OVA.GFP-transduced mice fed cuprizone for 6 weeks. (**E**) Representative images of the same approach used in **D** in mice transduced with AAV.Syn.GFP. Boxes in **D** and **E** indicate insets (**D**, i–iv; **E**, i–ii) shown at higher magnification immediately below the corresponding panel. (**F**) Optical sections from a *z*-stack image of a brain-infiltrating RFP^+^ OT-I T cell (red) in apposition to a GFP^+^ axon (green) in a mouse transduced with AAV.Syn.OVA.GFP and fed cuprizone for 6 weeks prior to adoptive transfer. (**G**) 3D renderings of an RFP^+^ OT-I T cell in contact with a GFP^+^ axon. (**H**) Schematic of the multichambered microfluidic device used to introduce T cells to axons. Representative image showing GFP^+^ axons under attack by RFP^+^ OT-I T cells; box indicates inset at higher magnification. (**I**) Axons (green) from cortical neurons transduced with AAV.Syn.OVA.GFP or AAV.Syn.GFP were stimulated for 72 hours with IFN-γ prior to incubation for 1 hour (top row) or 24 hours (bottom row) with RFP^+^ OT-I T cells isolated from the brain (BILs) 5 days after adoptive transfer into AAV.Syn.OVA.GFP-transduced mice fed cuprizone for 6 weeks. (**J**) The same approach as in **I** using blood-derived OT-I T cells. (**K**) Quantitation of axonal injury from the experiment shown in **I** after 24 to 72 hours. Low-magnification images in **A**, **B**, **D**, **E**, and **H** were acquired with 10× objective. High-magnification insets were acquired with 60× objective (**A** and **D**–**G**) or 40× objective (**H**). Immunostained tissue sections representative of *n* = 3–5 mice per condition. In vitro data representative of T cells independently isolated from *n* = 5 mice. Error bars show the 95% CI. **P < 0.01; ***P < 0.001; ****P < 0.0001 by unpaired, 2-tailed *t* test (**C**) or 1-way ANOVA with Dunnett’s multiple-comparison test (**K**).

B6 mice were transduced by intracranial inoculation of AAV.Syn.OVA.GFP, fed cuprizone or control chow for 6 weeks, irradiated, and adoptively transferred with OT-I or B6 CD8^+^ T cells. At 5 days after transfer, brain sections were analyzed by immunostaining to assess axonal injury. Cuprizone-demyelinated mice receiving OT-I T cells exhibited increased accumulation of axonal amyloid precursor protein (APP) (Figure 6A and Supplemental Figure 10). We also observed increased expression of nonphosphorylated neurofilament (npNF; SMI-32) (Figure 6B) and profound loss of GFP^+^ axons in fiber tracts such as the corpus callosum (Figure 6B) in cuprizone-demyelinated mice receiving OT-I T cells by adoptive transfer (Supplemental Figure 11). While it is possible that a reduction in axonal diameter associated with reduced levels of neurofilament phosphorylation may have contributed to the observed loss of GFP^+^ axons, quantitative analysis revealed that cuprizone-demyelinated mice receiving OT-I T cells had a substantial reduction in healthy axons, increased APP^+^ inclusions, and increased blebbing, relative to levels measured in mice receiving T cells that were not antigen specific (Figure 6C). To localize effector T cells, B6 mice were transduced by intracranial inoculation with AAV.Syn.OVA.GFP or AAV.Syn.GFP, fed cuprizone for 6 weeks, irradiated, and adoptively transferred with red fluorescent OVA-specific CD8^+^ T cells isolated from OT-I × RFP donors (OT-I.RFP). Microscopy revealed RFP^+^ cells in association with GFP^+^ axons in the cortex and corpus callosum (Figure 6D and insets). In mice transduced with AAV.Syn.GFP, only scarce RFP^+^ cells were observed (Figure 6E and insets). High-magnification *z*-stacks (Figure 6F) and 3D projections (Figure 6G) revealed RFP^+^ OT-I T cells physically associated with GFP^+^ axons exhibiting blebbing indicative of injury. Finally, to directly demonstrate axonal injury mediated by antigen-specific CD8^+^ T cells, we cultured primary murine cortical neurons in a multichambered microfluidics platform that allowed axons to grow into and through a channel that permitted introduction of T cells specifically to the axons en passant (Figure 6H). Neurons transduced with AAV.Syn.OVA.GFP or AAV.Syn.GFP and OT-I.RFP T cells from brain or blood were incubated with the axons. In the context of OVA expression, OT-I T cells elicited profound axonal injury within 1 hour (Figure 6, I and J, top row), whereas in the absence of OVA transduction such injury was not observed even at 24 hours (quantified in Figure 6K). These findings suggest that neuronal antigen–specific CD8^+^ T cells are recruited to the demyelinated CNS and directly mediate axonal injury.

### Neuronal MHC I and β2M expression is upregulated in MS patient brain tissue.

**Figure 7:**
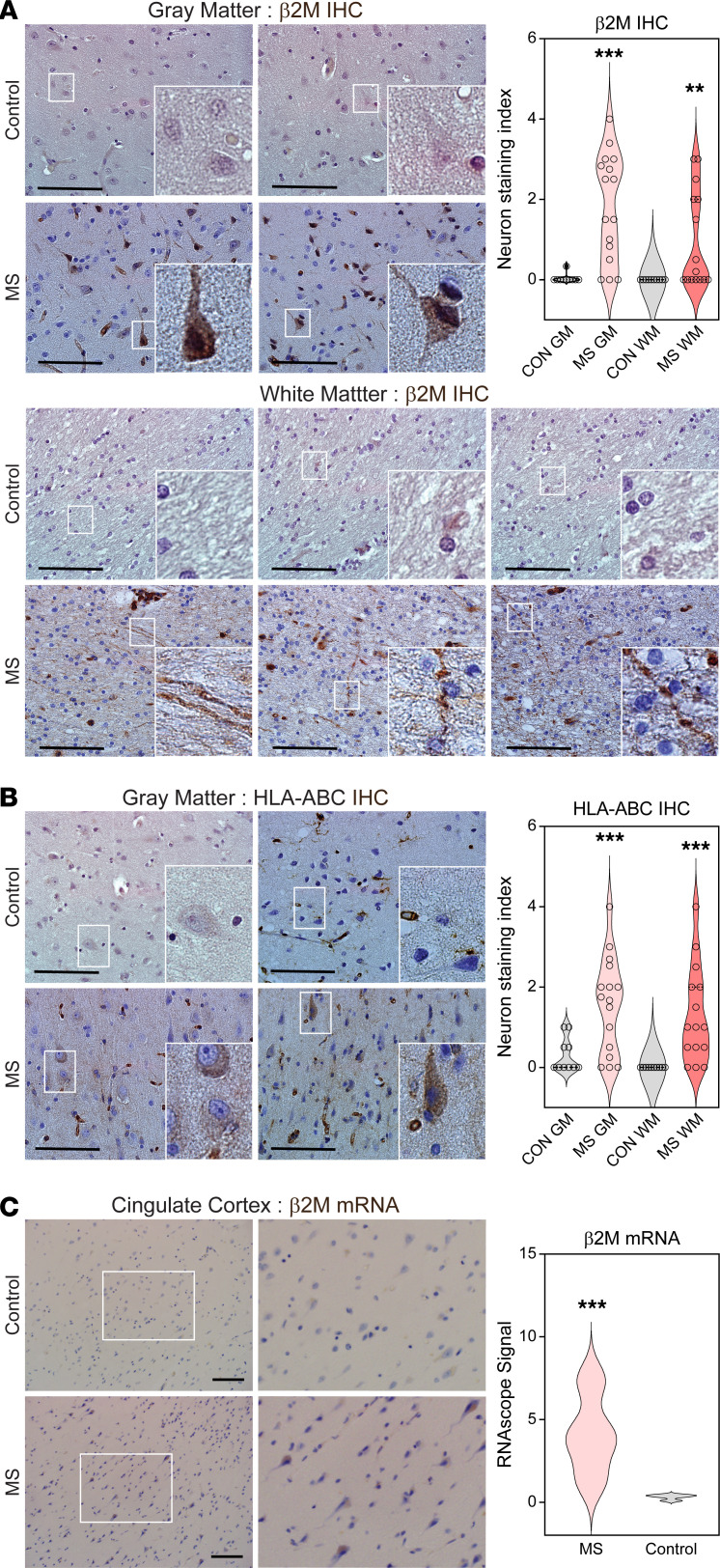
Neuronal MHC class I and β2M expression is upregulated in MS patient brain tissue. (**A** and **B**) Representative micrographs and quantitation of β2M (**A**) and HLA-A,B,C (**B**) immunostaining in cingulate cortical and thalamic gray matter (GM) and in adjacent white matter (WM) tracts from controls (CON) (*n* = 10) and MS patients (*n* = 18). (**C**) Representative micrographs and quantitation of in situ hybridization for β2M mRNA in MS and control cingulate cortex. Scale bars: 100 μm. Signal quantification shown in violin plots to the right of associated images. **P < 0.05; ***P < 0.01 by unpaired, 2-tailed *t* tests comparing MS vs. CON in GM and MS vs. CON in WM (**A** and **B**) or unpaired, 2-tailed *t* test (**C**).

In order to gauge the potential for CD8^+^ T cell–mediated targeting of MHC class I–positive neurons and axons in human disease, we immunostained MS and control brain tissues to compare levels of β2-microglobulin (β2M) and HLA-A,B,C expression in neurons in cortical gray matter (cingulate gyrus) and deep gray matter (thalamus). We found that in cingulate cortex from MS patients, neuronal cell bodies and axons in adjacent white matter tracks exhibited elevated levels of β2M (Figure 7A) and HLA-A,B,C (Figure 7B) compared with gray matter and white matter from controls. In controls, expression of β2M and HLA-A,B,C was restricted to endothelial cells and ramified microglia. As expected, HLA-A,B,C and β2M expression was found to correlate in normal-appearing gray matter and white matter from MS patients (Supplemental Figure 12). In situ hybridization similarly demonstrated increased neuronal expression of *b2M* mRNA in MS patient cingulate cortex (MS = 4.009 ± 1.085; control = 0.402 ± 0.036; Figure 7C) as well as in temporal cortex from patients with Alzheimer disease (AD = 1.921 ± 0.2769; control = 0.335 ± 0.168; Supplemental Figure 12), in line with recent reports implicating CD8^+^ T cells in the pathogenesis of AD (48–50). In contrast, we did not observe increased levels of mRNA for the immunoproteasomal subunit *PSMB8* (MS cingulate = 1.782 ± 0.599; control cingulate = 1.182 ± 0.226; AD temporal cortex = 1.617 ± 0.320; control temporal cortex = 0.511 ± 0.167; Supplemental Figure 12) or the antigen peptide transporter *TAP2* (MS cingulate = 0.458 ± 0.203; control cingulate = 0.993 ± 0.333; AD temporal cortex = 0.357 ± 0.054; control temporal cortex = 0.453 ± 0.098; data not shown) in either MS or AD brain tissue. These findings suggest that the disease state induces specific upregulation of the most distal components of the antigen presentation pathway in neurons. We further analyzed CD8^+^ T cell accumulation in normal-appearing white and gray matter from MS patients. We observed prominent infiltration of CD8^+^ T cells in normal-appearing white matter and occasional CD8^+^ T cell infiltrates in otherwise normal-appearing gray matter (Supplemental Figure 13). These cells were present in perivascular cuffs, meningeal compartments within sulci, and in white and gray matter parenchyma. Together, these data suggest that in addition to targeting axons within lesions in MS patients, CD8^+^ T cells may target MHC class I–expressing neuronal cell bodies located in gray matter tissues and nonlesional white matter distal from the site of lesions.

## Discussion

We show that a CNS neuron–restricted antigen is sampled by the peripheral immune system and that axons and neurons presenting this antigen on MHC class I are targeted by antigen-specific CD8^+^ T cells within the context of demyelination. This is in line with reports using Orex-HA mice in which orexin-HA fusion protein is produced by orexigenic neurons. In these studies, resting memory CD8^+^ T cells targeting HA in orexigenic neurons accumulated in the hypothalamus and contributed to injury (51, 52). Similar results were found in CamK-HA mice, where vasopressin-producing neurons of the hypothalamus were injured by CD8^+^ T cells despite widespread HA expression throughout the neuraxis (53). This was attributed to high basal MHC class I expression in the hypothalamus and the unique fenestrated blood-brain interface in this region of the CNS. In contrast to such basal expression and limited spatial effect, we provide evidence that mature cortical neurons upregulate antigen-processing genes and express MHC class I molecules on the axolemma in response to demyelination induced by either EAE or cuprizone. We also show that these axonal MHC class I molecules are competent to present neuron-specific peptide antigens. Our findings further indicate that CD8^+^ T cells directed against a neuron-specific antigen surveil the brain under steady-state conditions but do not accumulate and do not elicit injury until demyelination is induced. We show that CD8^+^ neuronal antigen–specific T cells, which we refer to as “nasT” cells, acquire an activated effector phenotype in demyelinated mice, are associated with demyelinated axons expressing cognate antigen on MHC class I, and directly injure such axons. Notably, we also provide evidence that MHC class I presentation is increased in neurons and axons in the MS brain, in agreement with a previous report that primarily focused on white matter lesions (54).

We have previously shown in several animal models that demyelinated axons are injured by CD8^+^ T cells (27–30, 55). Specifically, we demonstrated in the Theiler’s murine encephalomyelitis virus model that spinal cord–infiltrating CD8^+^ T cells injure demyelinated axons via a perforin- and granzyme B–dependent but viral antigen–independent mechanism. In these studies, adoptive transfer of anti-axonal CD8^+^ spinal cord–infiltrating lymphocytes from a neurologically impaired donor into a demyelinated but unimpaired host resulted in rapid paralysis and profound loss of spinal cord axons, supporting the idea that demyelination is necessary but not sufficient for loss of neurologic function. We also determined in an in vitro model that IFN-γ–induced presentation of neuron-intrinsic antigens on MHC class I renders axons vulnerable to perforin- and granzyme B–dependent injury mediated by antigen-specific CD8^+^ T cells (41). In line with these previous findings, we have hypothesized that a population of clonally expanded CD8^+^ T cells present in the demyelinated lesions of patients with MS are specific for neuronal antigens and directly injure demyelinated axons, leading to permanent loss of neurologic function and MS disease progression (42). Our current findings suggest that demyelination alone is sufficient to drive neuronal and axonal expression of MHC class I molecules loaded with self-antigen. Surprisingly, we also found upregulation of MHC class II transcripts by neurons (Supplemental Figure 1). While this finding may indicate either unexpected contamination of the response by microglial RNA or a microglial response to retrograde neuronal stress, the expression of MHC II on nonprofessional antigen-presenting cells is in line with a previous report of MHC II upregulation on oligodendrocyte progenitor cells (56). While beyond the scope of the current study, future exploration of the entire retrograde transcriptional program induced in neurons by distal inflammation and demyelination may reveal unique insights into pathogenic processes, including both novel immunological processes and stress responses that may confer heightened sensitivity to neuronal cell death.

Despite our demonstration that demyelination induced via 2 very different mechanisms drove upregulation of neuronal antigen processing and presentation mechanisms in the cortex and retina, it is possible that this response was caused by factors secondary to MOG EAE or cuprizone toxicity, rather than directly by demyelination, per se. For example, cuprizone is a copper chelator that has pleiotropic systemic effects, and cuprizone intoxication may have altered metabolic pathways that resulted in neuronal stress responses. Likewise, while induction of MOG EAE results in prominent white matter injury mediated by anti-myelin effectors, the model is associated with widely disseminated neuroinflammation (57, 58) that may have altered neuronal gene expression. However, whether a direct effect of myelin loss via the induction of pathogenic electrophysiological and metabolic processes or an indirect effect of the metabolic or neuroinflammatory stress induced by the demyelinating insult, our findings indicate that the demyelinated CNS creates an environment that drives neuronal and axonal MHC class I presentation of self-peptides, the recruitment and retention of neuronal antigen–specific CD8^+^ T cells, and injury and loss of axons. These events do not occur in mice that are intoxicated with cuprizone but not transduced with neuron-specific OVA, even when OT-I T cells are present. They do not occur in cuprizone-intoxicated mice transduced with neuron-specific OVA in the absence of OT-I T cells. And they do not occur in healthy, normally myelinated mice transduced with neuron-specific OVA even when OT-I T cells are present. Therefore, regardless of the direct versus indirect nature of the insult, the demyelinated brain is uniquely susceptible to injury mediated by CD8^+^ T cells directed against neuron-specific antigens.

The use of irradiation to “make space” in our model is another potential secondary factor that may have unmasked effects on local antigen presentation and antigen drainage from the CNS to the CLNs. Whole-body irradiation may have also induced neuroinflammation that was not directly associated with demyelination, possibly confounding the interpretation of our results. However, neuronal expression of antigen processing and presentation genes in both the cuprizone and EAE models and in both the cortex and the retina occurred in the absence of irradiation (Figure 1), indicating that demyelination itself was sufficient to drive this response. Similarly, irradiation was not employed in the experiments showing that demyelination was necessary and sufficient to induce presentation of SIINFEKL on axonal H2-K^b^ (Figure 2). Irradiation was also not a factor in the experiments showing that CNS transduction with AAV.Syn.OVA.GFP led to peripheral proliferation and activation of adoptively transferred OT-I T cells and to drainage of OVA-derived antigens to the deep CLNs in demyelinated mice (Figure 3). Irradiation did facilitate peripheral expansion of the adoptively transferred OT-I T cells, increasing the representation of these cells from 7% in nonirradiated hosts receiving the transferred cells to more than 70% in irradiated hosts (Supplemental Figure 8). However, despite this effect on peripheral expansion of the OT-I effector cells, irradiation did not alter the number or percentage of T cells infiltrating the brain in demyelinated mice (Supplemental Figure 9). So why did we use light irradiation for the adoptive transfer experiments looking at T cell activation and axonal injury in demyelinated hosts? An important perspective paper from Maine and Mule (59) regarding the “making room” or “Lebensraum effect” concept highlights the role of mild lymphopenia in lightly irradiated hosts in facilitating not only expansion of adoptively transferred T cells but also the impact of this homeostatic proliferation on maintenance of memory and effector phenotypes, increased sensitivity to antigen stimulation, and increased effector function. One potential key element facilitating this effect is irradiation-induced elimination of CD4^+^CD25^+^ regulatory T cells. Given that our adoptive transfer hosts were normal B6 mice (rather than immunodeficient hosts), relieving the inhibitory influence of host regulatory T cells, in addition to “making space” for expansion, was considered important to accelerating the evolution of pathogenesis in our model. Our unpublished observations indicate that CNS transduction with AAV.Syn.OVA.GFP and demyelination with cuprizone in OT-I mice leads to profound axonal and neuronal injury without the need for either adoptive transfer or irradiation. However, within the context of the current study we were seeking to maximize the ability to control critical variables (demyelinated vs. myelinated, AAV.Syn.OVA vs. AAV.Syn.GFP, OT-I vs. B6 T cell adoptive transfer) and elicit pathogenesis within a circumscribed timeline. So, does the irradiation compromise our findings? A study from the Rivest group (60) suggests that this manipulation did not alter the CNS in a manner that would impact our study. These investigators used whole-body irradiation with a lethal dose of 10 Gy (vs. non-lethal 4 Gy dose in our model) to myeloablate 3-month-old B6 mice. They found that even with this lethal dose there was only transient evidence of neuronal injury (undetectable by 24 hours after irradiation) and there was no persistent effect on neuroinflammation. Likewise, integrity of the blood-brain barrier was not affected by lethal irradiation. Given the lower dose of radiation used in our model, we conclude that making space in the adoptive transfer hosts likely did not impact the CNS in a manner that compromises our interpretations. Moreover, even if some aspect of the irradiation did alter the model in some unexpected manner, our observations still indicate that the demyelinated brain is uniquely susceptible to injury by CD8^+^ T cells directed against neuron-specific antigens.

While AAVs are nonreplicating and hypoimmunogenic, it is possible that the viral vector we employed elicited danger signals that enhanced immunity generated against peptide epitopes, resulting in a nonphysiological effect in our model. Similarly, the injury associated with intracranial delivery of the vector may have elicited local neuroinflammation that enhanced T cell recruitment or effector functions. However, we intentionally transduced the CNS with AAV 2 weeks prior to initiating the cuprizone diet to avoid these issues. Moreover, the OT-I T cells were not introduced into hosts until 6 weeks after initiation of cuprizone and therefore 8 weeks after delivery of the AAV. Given that we observed essentially no T cell recruitment or neural injury in mice transduced with AAV.Syn.GFP and we observed only limited infiltration of antigen-specific T cells in AAV.Syn.OVA.GFP-transduced mice that were not demyelinated, it is likely that our model was not impacted by these issues.

Another potential confounder is the fact that AAV-mediated transduction of chicken OVA results in neuron-specific expression of a protein that has not undergone central tolerance or substantial peripheral tolerance. In addition, OT-I TCR-transgenic T cells have elevated basal activation relative to endogenous T cells in B6 mice (unpublished observations) and have unusually strong TCR affinity for SIINFEKL in the context of H2-K^b^ presentation (61). However, while our model system does not fully recapitulate tolerance breaking against endogenous CNS neuron–restricted antigens, the OT-I system has been utilized to interrogate afferent and efferent immunity when OVA expression is restricted to other CNS cell types, both in the context of CNS infection and demyelinating disease (46, 47, 62–64). Of note, these studies utilized EAE to determine whether sentinel CD4^+^ and CD8^+^ T cells are recruited to the demyelinated and inflamed CNS, a system that may be complicated by the profound neuroinflammatory state associated with EAE. We chose to use the cuprizone model in order reduce these effects (65). Indeed, this model allowed us to discover that neuronal antigen–specific T cells surveil but do not accumulate in the healthy CNS and that these cells robustly accumulate in the demyelinated CNS even in the absence of the intense neuroinflammation that occurs in EAE.

Finally, while our findings indicate that CD8^+^ T cells directed against neuron-specific antigens can mediate axonal injury in the context of demyelination, it remains unknown whether such antigen-specific T cells are present in patients with MS or other CNS autoimmune diseases. The identity of potential neuron-specific autoantigens in MS patients remains unknown and the mechanisms by which tolerance to such antigens could be broken in these patients are unclear. Some have conjectured that the broad immune response to myelin epitopes in patients with MS may preclude the identification of so-called “cryptic” neuronal autoantigens. This may explain why such targets have not been identified to date using traditional methods (66). However, while the heterogeneity of MS may challenge the discovery of neuron-specific autoantigens and autoreactive effector CD8^+^ T cells, this same variability also suggests that a specific subset of MS patients — perhaps those with certain primary or secondary progressive features — may harbor pathogenic neuronal antigen–specific T cells. Indeed, this model of CD8^+^ T cell–driven injury is strongly supported by prior reports of clonally expanded CD8^+^ T cells in MS lesions as well as the evidence we provide of elevated levels of MHC class I and β2M in neurons and axons of MS patients. Of note, HLA-A,B,C^+^ and β2M^+^ neurons were found in gray matter, with no evidence of local leukocyte infiltration or gray matter demyelination. It is therefore possible that this expression was induced in response to distal cues elicited within demyelinating lesions that traveled retrogradely through the axon, as we have previously reported (40), or in response to demyelination induced by upstream deafferentation. Future work should focus on identifying which factors drive this response, whether they signal retrogradely or anterogradely, and whether this signaling persists in chronic active or inactive lesions. Future work should also investigate neuronal expression of antigen-processing genes, such as *PSMB8* and *TAP2*, during acute inflammatory demyelination. As we did not detect upregulation of these genes in cortical neurons from MS patient autopsy tissue, it is possible that they are only transiently upregulated (as we have noted in response to IFN-γ signaling; ref. 40), that these mRNA molecules are transported into the axon and only translated locally at the site of peptide presentation (67), or that neurons are limited to TAP1/2-independent MHC class I peptide presentation mechanisms (68, 69).

In summary, our findings have important implications for the understanding of pathogenic mechanisms in progressive MS and for the development of therapeutic strategies to prevent disease progression. We demonstrate that neuron-restricted antigens are sampled by the peripheral immune system and show that demyelination alone is sufficient to drive upregulation of peptide-loaded MHC class I on neurons and denuded axons, rendering them susceptible to immunological attack by cognate CD8^+^ T cells. Immune targeting of neuronal antigens in MS is consistent with the loss of axons observed in white matter lesions and with the diffuse loss of neurons and synapses in otherwise normal appearing gray matter. Pathogenic specificity is suggested by the absence of such findings in other demyelinating diseases, such as neuromyelitis optica and MOG antibody disease (70). Mechanistically, in addition to providing a direct target for CD8^+^ T cell–mediated axonal injury within demyelinated white matter lesions, the upregulation of MHC class I on neurons in gray matter may also provide an initial stimulus to surveilling CD8^+^ T cells that drives proinflammatory cytokine secretion and further immune cell recruitment, precipitating diffuse neuron loss or the formation of gray matter lesions. The concept of anti-neuronal CD8^+^ T cell–initiated gray matter injury is supported by the evidence we provide of increased neuronal HLA-A,B,C and β2M expression in the gray matter of MS patients. Our findings provide a strong preclinical justification for follow-up studies investigating the pathogenic role of neuronal antigen–specific CD8^+^ T cells in patients with MS.

## Methods

### Mice.

C57BL/6 WT (stock 000664), B6.PL-*Thy1a*/CyJ (Thy1.1, stock 000406), B6.Cg-Tg(Syn1-cre)671Jxm/J (Syn.Cre, stock 003966), B6.129(Cg)-*Gt(ROSA)26Sor^tm4(ACTB-tdTomato,-EGFP)Luo^*/J (mT/mG, stock 007676), B6J.129(Cg)-*Rpl22^tm1.1Psam^*/SjJ (Rpl22, stock 029977), and C57BL/6-Tg(TcraTcrb)1100Mjb/J (OT-I, stock 003831) TCR-transgenic mice with SIINFEKL:H2-K^b^–restricted CD8^+^ T cells were obtained from the Jackson Laboratory. OT-I mice were crossed with homozygous Thy1.1^+/+^ or mT/mG^+/+^ mice to generate OT-I.Thy1.1^+/–^ or OT-I.mT/mG^+/–^mice used for adoptive T cell transfer experiments. Rpl22 mice were crossed with Syn.Cre mice to generate neuron-specific HA-tagged polyribosomal RNA for microarray analysis. All F_1_ offspring used in experiments were screened for TCR (Vα2Vβ5) and RFP or Thy1.1 transgene expression by flow cytometry on immune cells isolated from blood. Male and female mice between 6 and 10 weeks of age at the start of experiments were used for all mouse studies.

### Cuprizone, EAE, and pertussis toxin injections.

EAE was induced as previously described (57, 71, 72) using MOG_35–55_ peptide (Hooke Laboratories EAE induction kit, EK-2110). Pertussis toxin (200 ng/mouse, i.p.; Hooke Laboratories) was injected on day 0 and 2 relative to immunization. Only animals exhibiting clinical disease were examined by transcriptional analysis. For experiments modeling blood-brain barrier disruption, 200 ng pertussis toxin was injected i.p. in 500 μL PBS as above at 96 hours and 48 hours prior to adoptive transfer of CD8^+^ T cells. Cuprizone (bis[cyclohexanone]oxaldihydrazone; Sigma-Aldrich) diet containing 0.3% w/w cuprizone was prepared by Test Diet in 5LG6 base diet and utilized within 6 months of manufacture. Mice were allowed access to experimental or control (5LG6) diet ad libitum and monitored weekly for 6 weeks prior to further manipulation. For intracerebral injections, anesthetized mice were injected with 1.5 μL containing 3 × 10^9^ genomic copies (gc) of AAV1.Syn.OVA.GFP or AAV1.Syn.GFP at 0.5 μL/min.

### RiboTag translational profiling.

SynCre.Rpl22 mice were perfused transcardially with cold PBS containing 20 μg/mL cycloheximide and 0.1 mg/mL heparin and subsequently processed as previously described to isolate neuronal ribosome-bound mRNA (43). Mouse cortex was grossly dissected and homogenized in buffer containing 45 mM Tris, 100 mM KCl, 12 mM MgCl_2_, 1% NP-40, 20 μg/mL cycloheximide, 1 mg/mL heparin, 0.5% RNasin (Promega), 1% protease inhibitor cocktail (Sigma-Aldrich), and 1 mM DTT. Polyribosomes were captured using 7.5 μg/mL anti-HA1.1 monoclonal antibody (16B12; BioLegend, 901501) and mRNA was eluted off the bead-bound ribosomes using Buffer RLT Plus (Qiagen) and RNeasy Micro Plus spin column assemblies (Qiagen).

### T cell transfers and labeling.

CD8^+^ T cells were magnetically purified (Miltenyi Biotec, 130-095-236) from lymph nodes of B6 or OT-I mice and resuspended in 50% FBS/ PBS at 1 × 10^7^ cells per mL for i.v. injection. Recipient mice were irradiated with 4 Gy 4 hours prior to i.v. injection of 200 μL containing 2 × 10^6^ CD8^+^ T cells.

### Patient samples.

Paraffin-embedded postmortem brain tissue sections were obtained from the Mayo Clinic Tissue Registry, Mayo Clinic Alzheimer’s Disease Research Center, Normal Aging Brain Collection Amsterdam, and the Netherlands Brain Bank (Supplemental Table 3). For immunohistochemistry, we analyzed samples from controls (*n* = 6 females and *n* = 4 males) aged 66 *±* 6 (mean *±* SD) years of age and samples from secondary progressive MS patients (*n* = 16 females and *n* = 2 males) aged 66 ± 7 years of age. Immediately prior to immunostaining or in situ hybridization, 10-μm-thick sections were prepared by the Mayo Clinic Pathology Research Core facility.

### Cortical neuron cultures and microfluidic devices.

Cortical neurons were prepared from C57BL/6 mouse embryonic day 15 (E15) pups as previously described (41, 72) and seeded at 4.5 × 10^5^ cells per cm^2^ (1 × 10^6^ cells per mL) on poly-ornithine–coated plates. In some experiments, cells were infected at plating with 2,000 MOI (2 × 10^9^ gc/mL) of AAVs. In some experiments, neurons were cultured in polydimethylsiloxane (PDMS) microfluidic axon isolation chambers, as previously described (41). The dimensions of these chambers are described elsewhere (40).

### Immunohistochemistry and in situ hybridization.

Tissue sections (10 μm thick) were immunostained with 5 μg/mL rabbit anti-β2M (13511-1-AP, Invitrogen), 1:200 mouse anti–pan-HLA-A,B,C (W6/32; Ebiosciences, 14-9983-82), 1:400 mouse anti–HLA-DR (LN3; Thermo Fisher Scientific, Ma5-11966), or 1:500 mouse anti-PLP (PLPc1; Bio-Rad, MCA839G) antibody. Automated in situ hybridization was performed by the Mayo Clinic Pathology Research Core following optimization and validation using the following RNAscope 2.5 LS Probes (ACD Bioteche) with RNAscope 2.5 Reagent Kit Brown (ACD Bioteche, 322100) in accordance with the manufacturer’s instructions: Hs-β2M (ACD Bioteche, 310168), Hs-TAP2 (ACD Bioteche, 494308), and Hs-PSBM8 (ACD Bioteche, 546838). Hs-PPIB (ACD Bioteche, 313908) and dap-B (ACD Bioteche, 312038) probes were used as positive and negative probes on all tissues to confirm RNA integrity and sample quality. For semiquantitative analysis of in situ hybridization, digital images were processed and analyzed using Fiji ImageJ software as previously described (73).

### Statistics.

All graphs show mean and 95% CIs unless otherwise indicated. *P* values less than 0.05 were considered significant. For multiple comparisons, 1-way or 2-way analysis of variance (ANOVA) or nonparametric (Kruskal-Wallis) tests were performed where appropriate. Reported *P* values were corrected for multiple comparisons (Holm-Šidák, Dunnett’s, or Benjamini-Hochberg correction for ANOVA; Dunn’s correction for Kruskal-Wallis; Benjamini-Hochberg for multiple unpaired *t* tests). Unpaired, 2-tailed Student’s *t* tests were used for comparisons made between 2 groups.

### Study approval.

All animal experiments were approved by the Mayo Clinic institutional animal care and use committee in accordance with the NIH *Guide for the Care and Use of Laboratory Animals* (National Academies Press, 2011). Study approval for human tissues was granted by the Mayo Clinic institutional review board (IRB 18-008128).

### Data availability.

All data are available in the main text or the supplemental material. Individual data points for each figure are available in the Supporting Data Values.xls file. MIAME-compliant microarray data are deposited with the NCBI Gene Expression Omnibus under accession number GSE241781. Please contact the author for any further material or data requests.

## Author contributions

CLH and BDSC conceptualized the study. BDSC, EMG, MMS, KM, LSM, MSW, and CLH developed the methodology. BDSC, EMG, MMS, KM, LSM, and MSW carried out the investigation. BDSC and CLH generated figures. CLH acquired funding, provided project administration, and supervised the project. BDSC and CLH wrote the original draft of the manuscript, which was reviewed and edited by BDSC, EMG, MMS, KM, LSM, MSW, and CLH.

## Supplementary Material

Supplemental data

Supporting data values

## Version 1 09/07/2023

In-Press Preview

## Version 2 11/01/2023

Electronic publication
