# Supplementary material for: CD8+ T cells recognizing a neuron-restricted antigen injure axons in a model of multiple sclerosis
Source: J Clin Invest. 2023 Nov 1;133(21):e162788. doi: 10.1172/JCI162788 (PMC10617772; doi:10.1172/JCI162788)
Supplement: Supplemental data [file jci-133-162788-s008.pdf]

Supplemental Materials

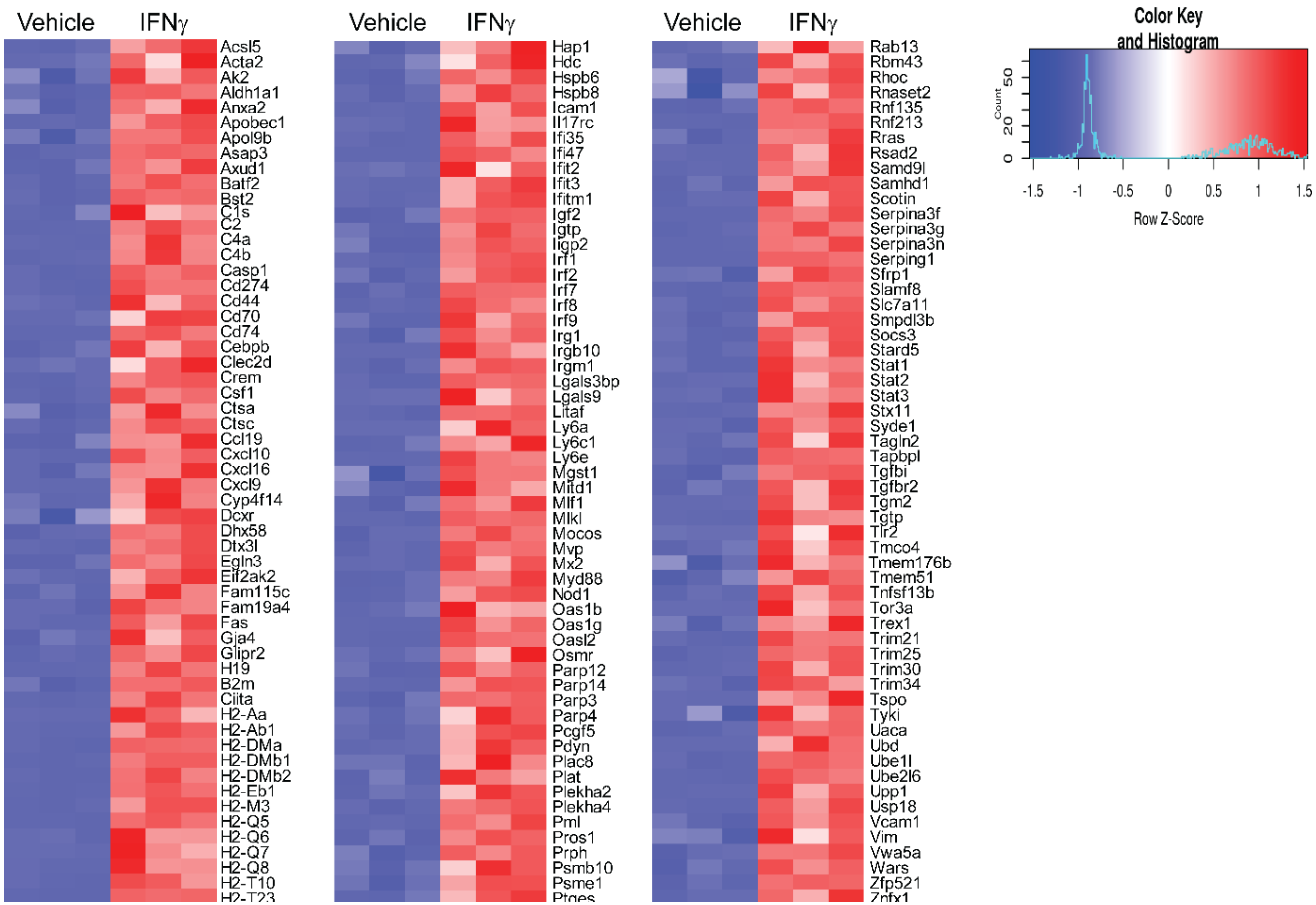

Supplemental Figure 1. Genes upregulated in neurons by axonal stimulation with IFNγ. Mouse cortical neurons were cultured in microfluidic chambers and allowed to elaborate axons into the distal chamber for 12-14 days. Axon fields were treated with 100 ng/mL IFNγ for 72 hours and then mRNA was isolated from the cell body chamber for microarray analysis. Heat map depicts Z scores for each gene across samples that were found to be significantly upregulated by IFNγ stimulation relative to vehicle control. (>2FC; P< 0.05).

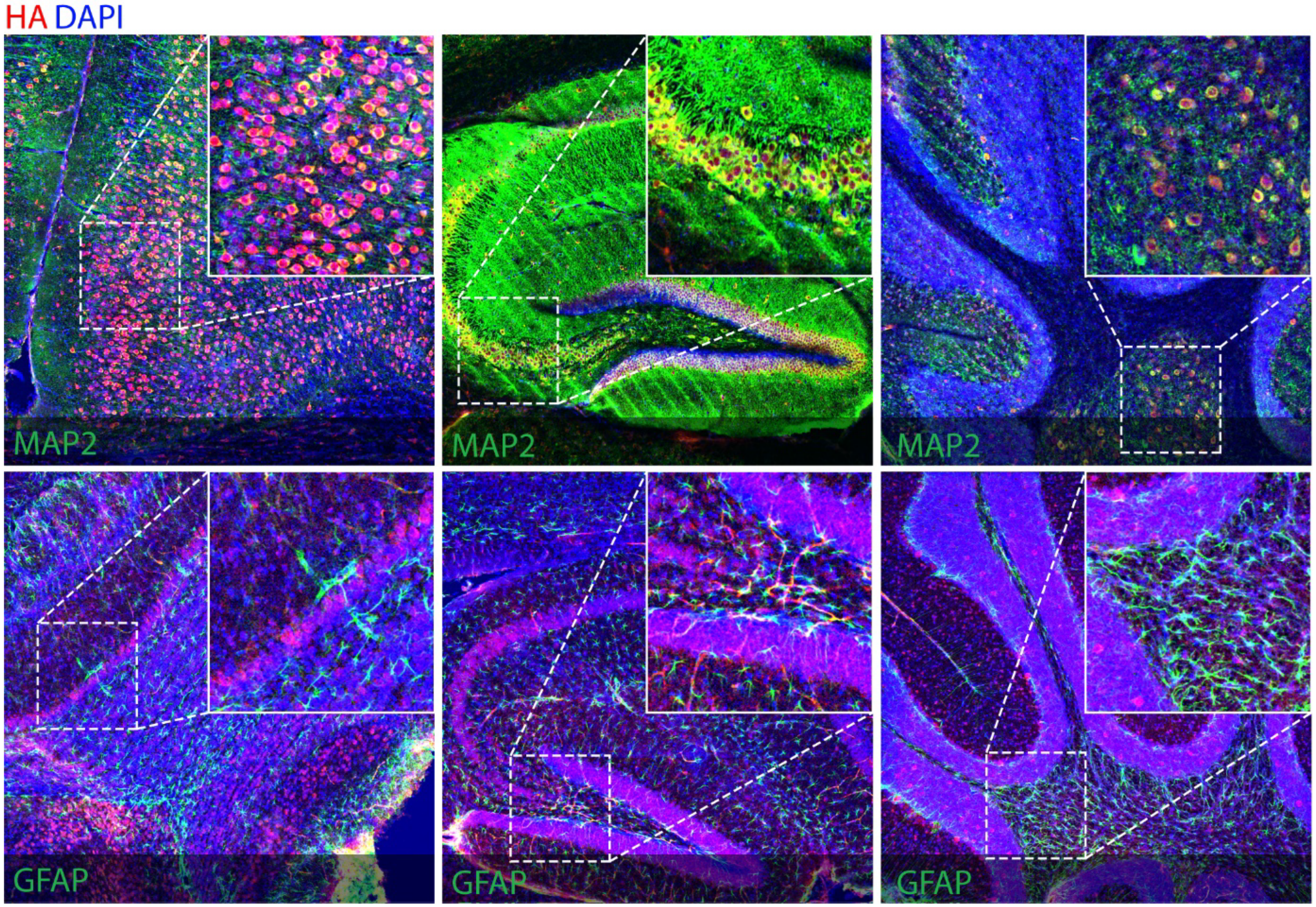

Supplemental Figure 2. Neuron-restricted expression of HA-tagged ribosomes in Syn-Cre.RPL22 mice. Representative micrographs (10x) of brain sections from Syn-Cre.RPL22 mice showing immunostaining for hemagglutinin (HA; red), the neuronal marker MAP2 (green; top row) and the astroglial marker GFAP (green; bottom row) in cortex (left), hippocampus (middle), and cerebellum (right). Digitally magnified insets of outlined areas are shown in white boxes.

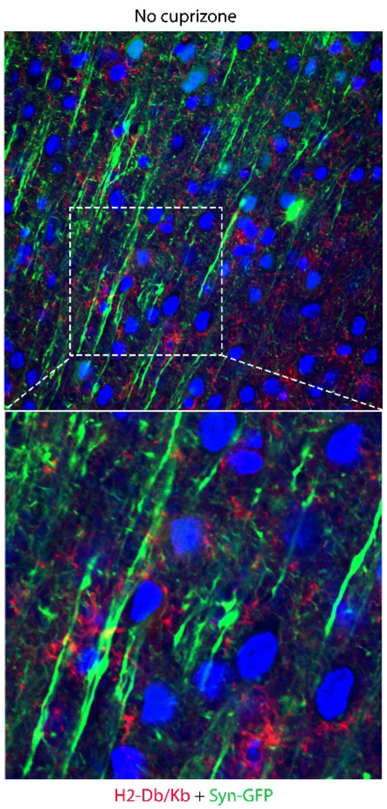

Supplemental Figure 3. Representative photomicrographs (40x magnification) from B6 mice on normal chow (no cuprizone) 8 weeks after intracranial inoculation with AAV1.Syn.OVA-GFP showing glial H2-D/K expression (red) near AAV1.Syn.OVA-GFP+ axons (green) that are negative for H2-D/K. This indicates that MHC class I is only upregulated on AAV1.Syn.OVA-GFP-transduced axons in the context of demyelination.

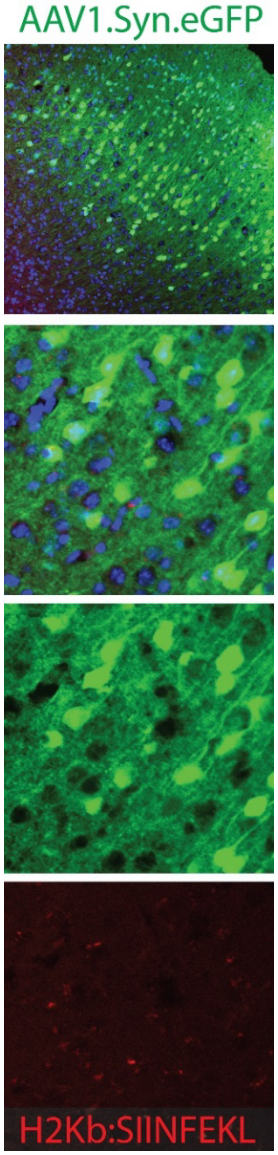

Supplemental Figure 4. Representative photomicrographs of AAV.Syn.GFP transduced mouse cortex showing absence of anti-H2Kb:SIINFEKL labeling (red) on GFP⁺ neurons and axons in mice demyelinated by cuprizone. Bottom 3 panels at higher magnification show merged image, GFP signal, and H2Kb:SIINFEKL signal, respectively. Compare to H2Kb:SIINFEKL staining on AAV.Syn.OVA.GFP-transduced cortical neurons in mice demyelinated by cuprizone (Figure 2D-E).

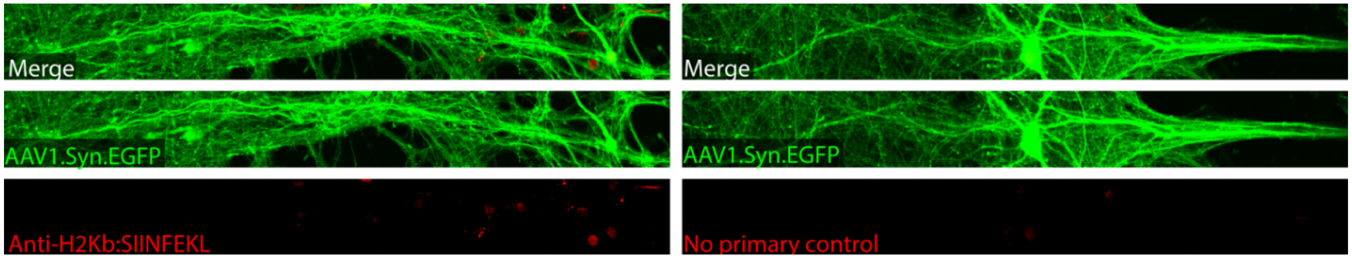

Supplemental Figure 5. (Left panels) Absence of H2Kb:SIINFEKL labeling (red) on AAV1.Syn.GFP-transduced (green) cortical neuron axons in microfluidic chambers following axonal stimulation with 100 ng/mL IFNγ for 72 hours. Compare to H2Kb:SIINFEKL expression shown on identically treated AAV1.Syn.OVA-GFP+ axons in Figure 2G. No primary control is shown on the right.

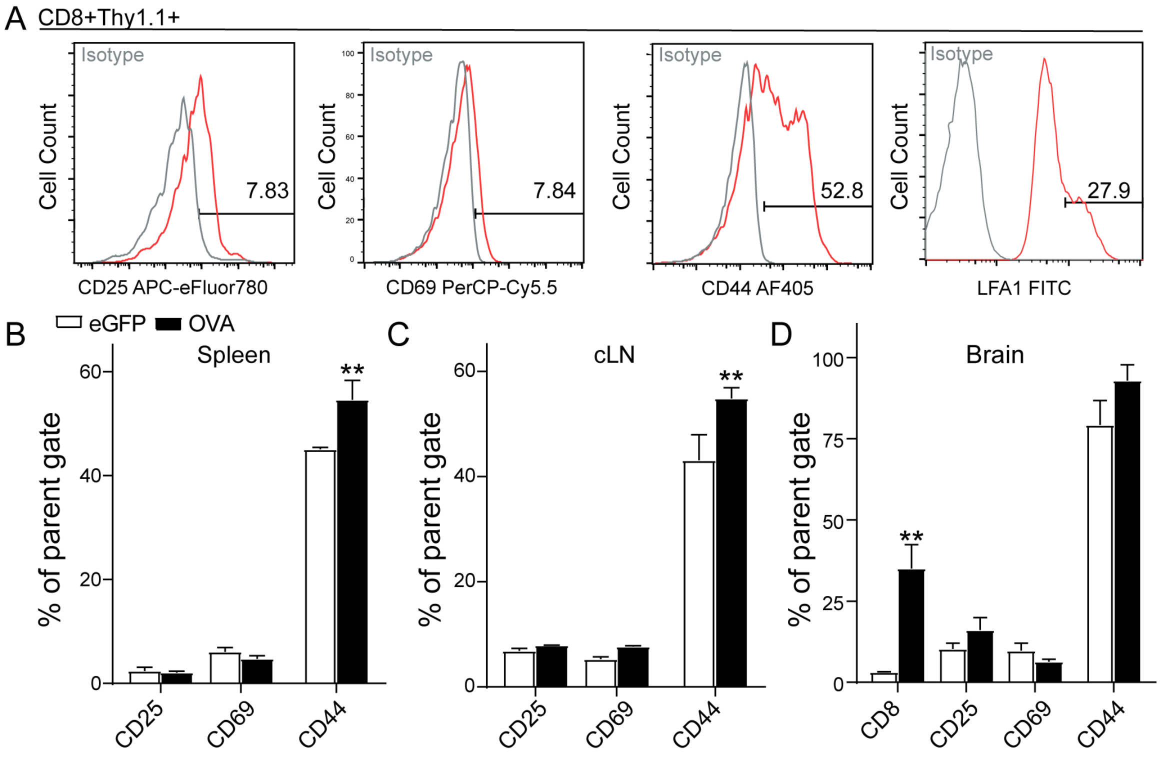

Supplemental Figure 6. Activation markers on OT-I T cells in spleen, deep cervical lymph nodes, and brain after transfer into cuprizone demyelinated mice. B6 mice were inoculated intracranially with AAV1.Syn.OVA-GFP (OVA) or AAV1.Syn.GFP (GFP) and 2 weeks later started on cuprizone diet. After 6 weeks on cuprizone, mice were adoptively transferred with $2x10^6$ $CD8^+$ T cells from OT-I.Thy1.1 donors. Spleen, deep cervical lymph nodes (cLN), and brain were collected 8 days after the transfer. **A**) Gating strategy for determining positivity for the indicated T cell activation markers on $CD8^+Thy1.1^+$ OT-I T cells. Quantitation for spleen (**B**), cLN (**C**), and brain (**D**). Means ± SEM are shown. **P< 0.01.

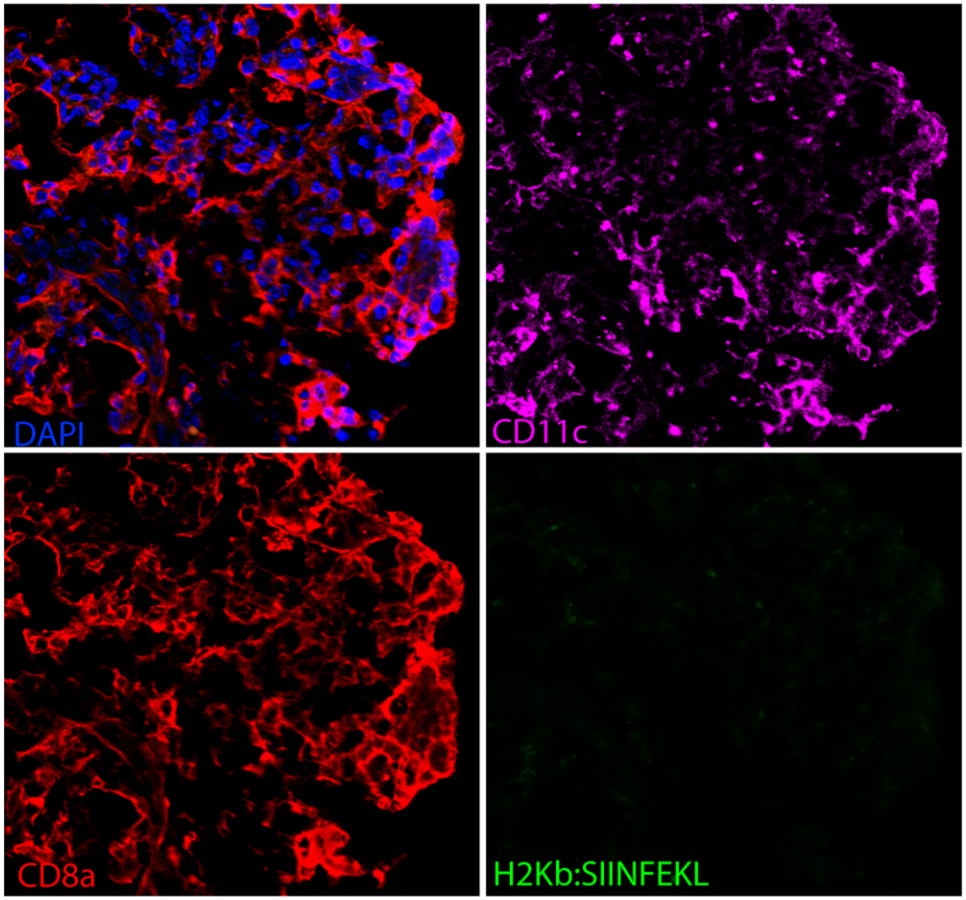

Supplemental Figure 7. Representative images (60x magnification) of immunostaining of deep cervical lymph nodes from cuprizone-fed mice transduced by intracranial inoculation with AAV.Syn.GFP. CD11c$^+$ dendritic cells (pseudocolored purple) and CD8a$^+$ T cells and dendritic cells (red) are shown; DAPI is shown in blue. No H2Kb:SIINFEKL staining (green) was detected. Compare to the H2Kb:SIINFEKL staining in deep CLN in demyelinated AAV.Syn.OVA.GFP mice provided in Figure 3E-H.

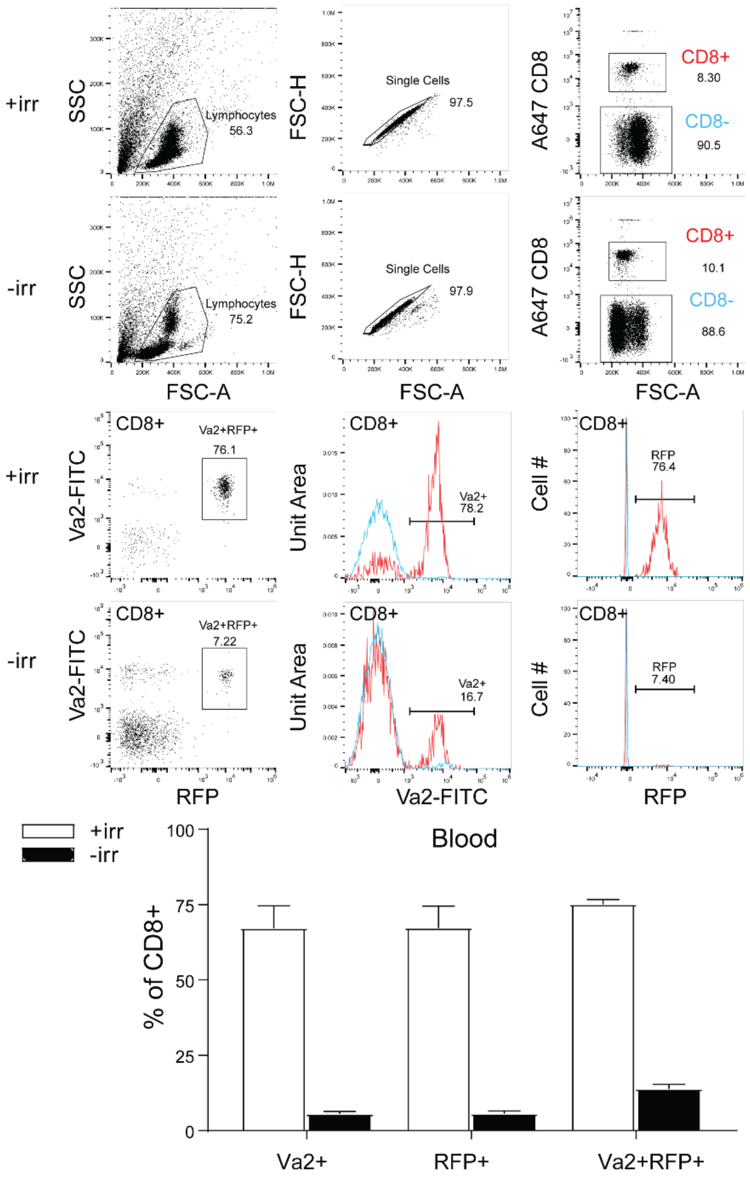

Supplemental Figure 8. B6 mice were inoculated intracranially with AAV1.Syn.OVA-GFP and 2 weeks later started on cuprizone diet. After 6 weeks on cuprizone, mice were injected i.v. with $2x10^6$ OT-I.RFP $CD8^+$ T cells. Recipient mice were either irradiated (400 rads) or left untreated 4 hours prior to adoptive transfer. Representative flow cytometry plots show circulating lymphocytes in irradiated (+Irr.) and nonirradiated (-Irr.) mice 8 days after transfer. Gating strategy is shown for live cells, single cells, and $CD8^+$ T cells. Percent of $CD8^+$ T cells staining positive for Vα2 and RFP is quantified below. Means ± SEM are shown.

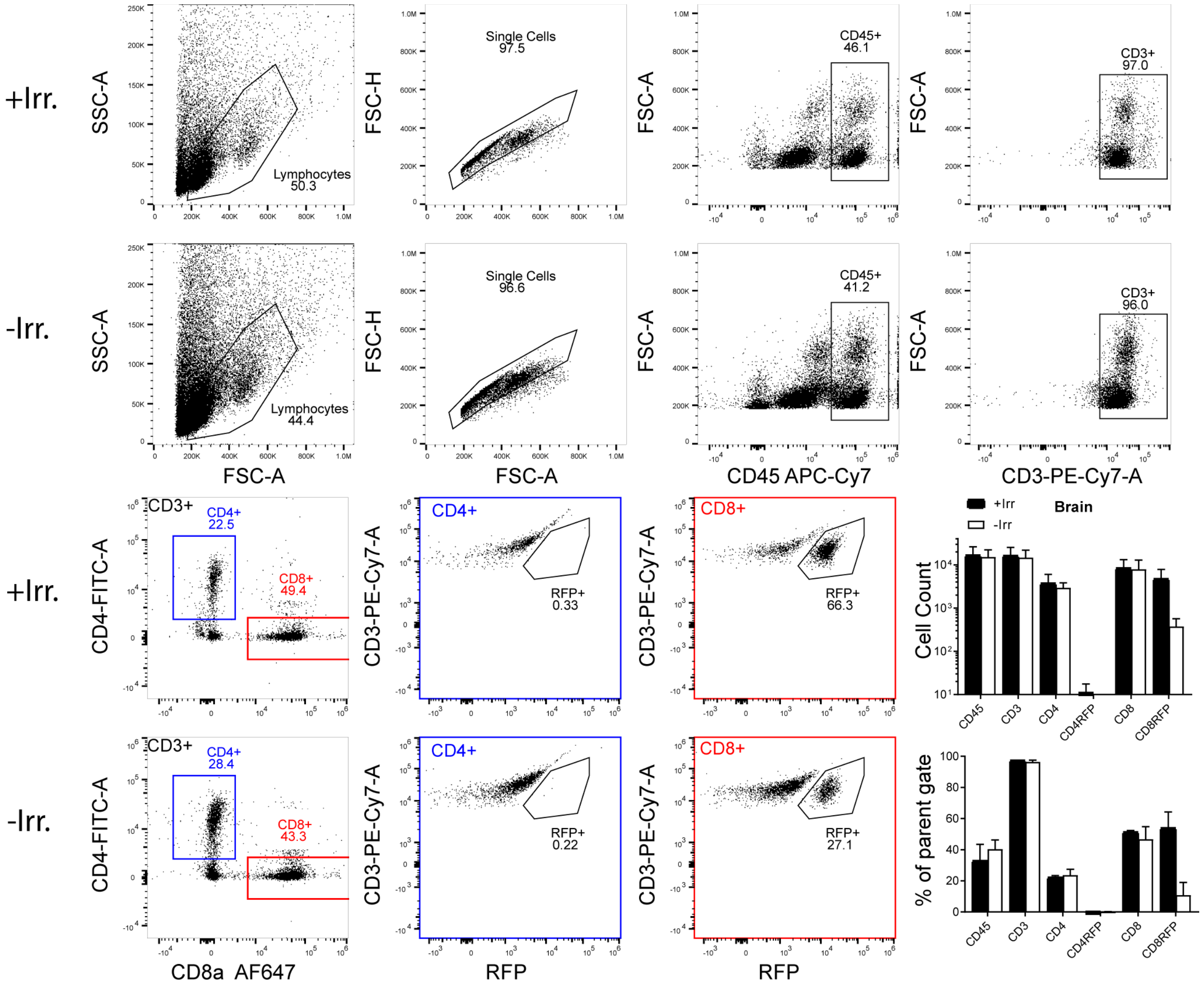

Supplemental Figure 9. B6 mice were intracranially inoculated with AAV1.Syn.OVA.GFP and 2 weeks later started on cuprizone diet. After 6 weeks, recipient mice were either irradiated (400 rads) or left untreated 4 hours prior to adoptive transfer mice by i.v. injection of 2x10^6 OT-I.RFP $CD8^+$ T cells. Representative flow cytometry plots show brain infiltrating lymphocytes in irradiated (+Irr.) and nonirradiated (-Irr.) mice 8 days post transfer. Gating strategy is shown for live cells, single cells, $CD45^+$ high (non-microglial) immune cells, $CD3^+$ (T cells), and $CD4^+$ and $CD8^+$ T cell subsets. As shown in supplemental Figure 8, non-irradiated recipients had far fewer circulating OT-I T cels post transfer. Yet, even in the absence of irradiation these cells still accumulated in the brain in AAV1.Syn.OVA.GFP transduced mice. This indicates irradiation is not necessary for T cell recruitment to the brain.

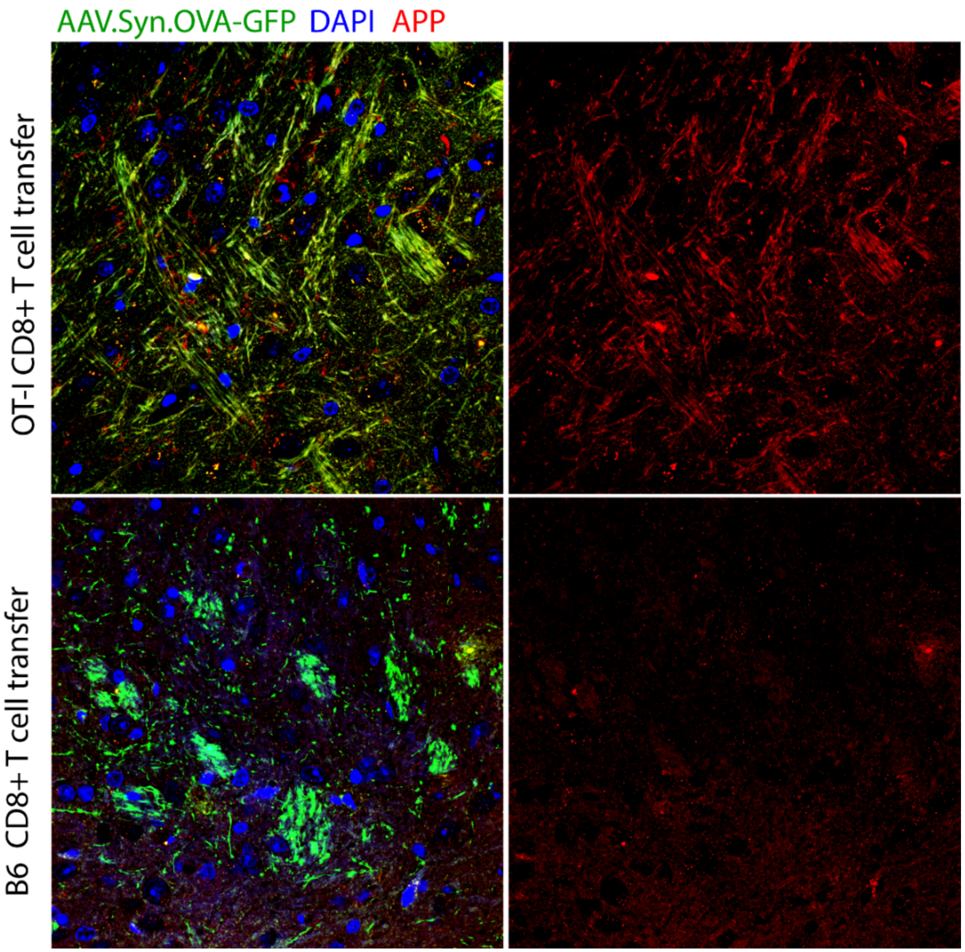

Supplemental Figure 10. Injury of OVA$^+$ axons following adoptive transfer of OT-I CD8$^+$ T cells into demyelinated mice. B6 mice were inoculated intracranially with AAV1.Syn.OVA-GFP and 2 weeks later started on cuprizone diet. After 6 weeks on cuprizone, mice were injected i.v. with 2x10^6 CD8$^+$ T cells from OT-I or B6 mice. Representative micrographs show amyloid precursor protein (APP) immunostaining on OVA-GFP$^+$ axons in cuprizone mice 8 days after receiving adoptive transfer of CD8$^+$ cells.

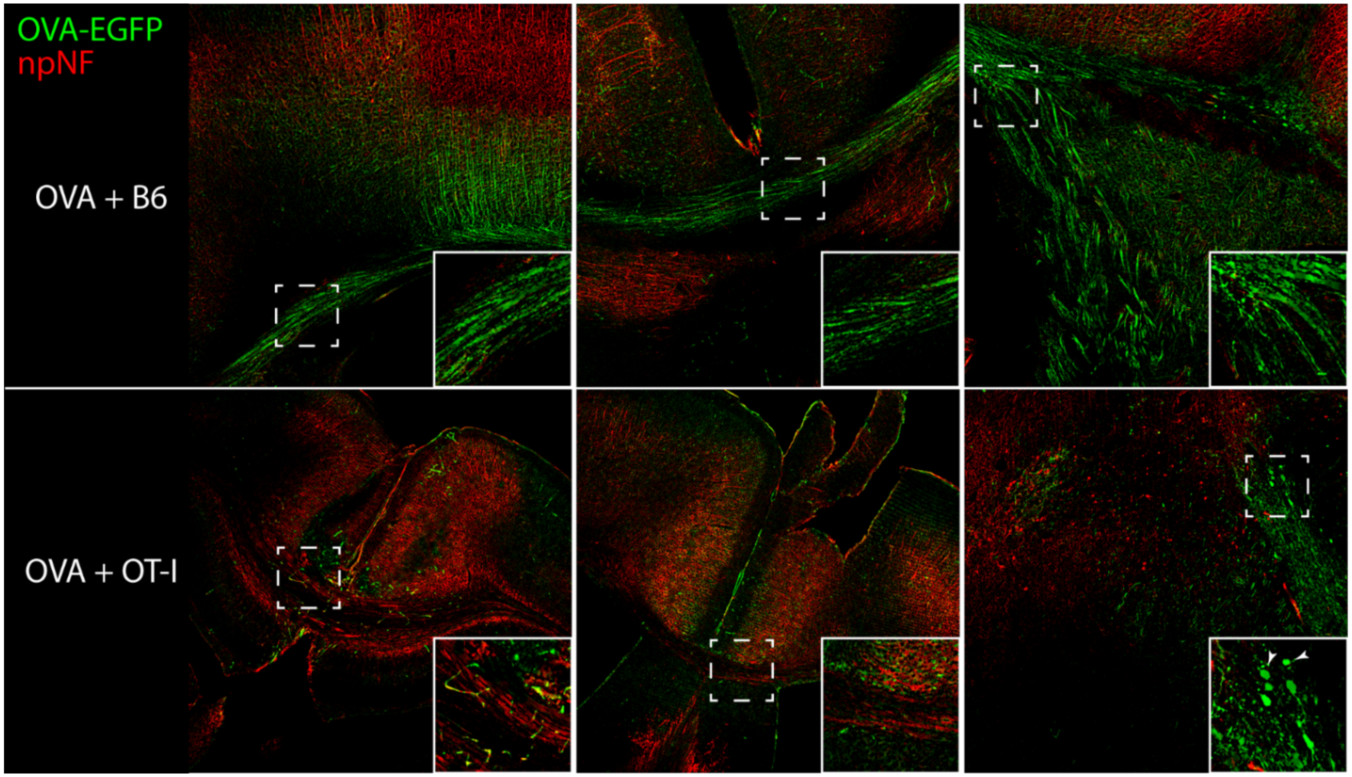

Supplemental Figure 11. Representative images showing non-phosphorylated neurofilament (npNF, SMI32; red) and GFP$^+$ axons (green) in the corpus callosum in AAV.Syn.OVA.GFP-transduced cuprizone-demyelinated mice receiving adoptive transfer of B6 or OT-I CD8$^+$ T cells. As shown, the GFP signal is substantially reduced in the corpus collosum and striatum of the OT-I recipient animals. While some GFP$^+$ neurons remain in the OT-I recipient, the morphology of these cells is indicative of ongoing injury (arrowheads bottom right).

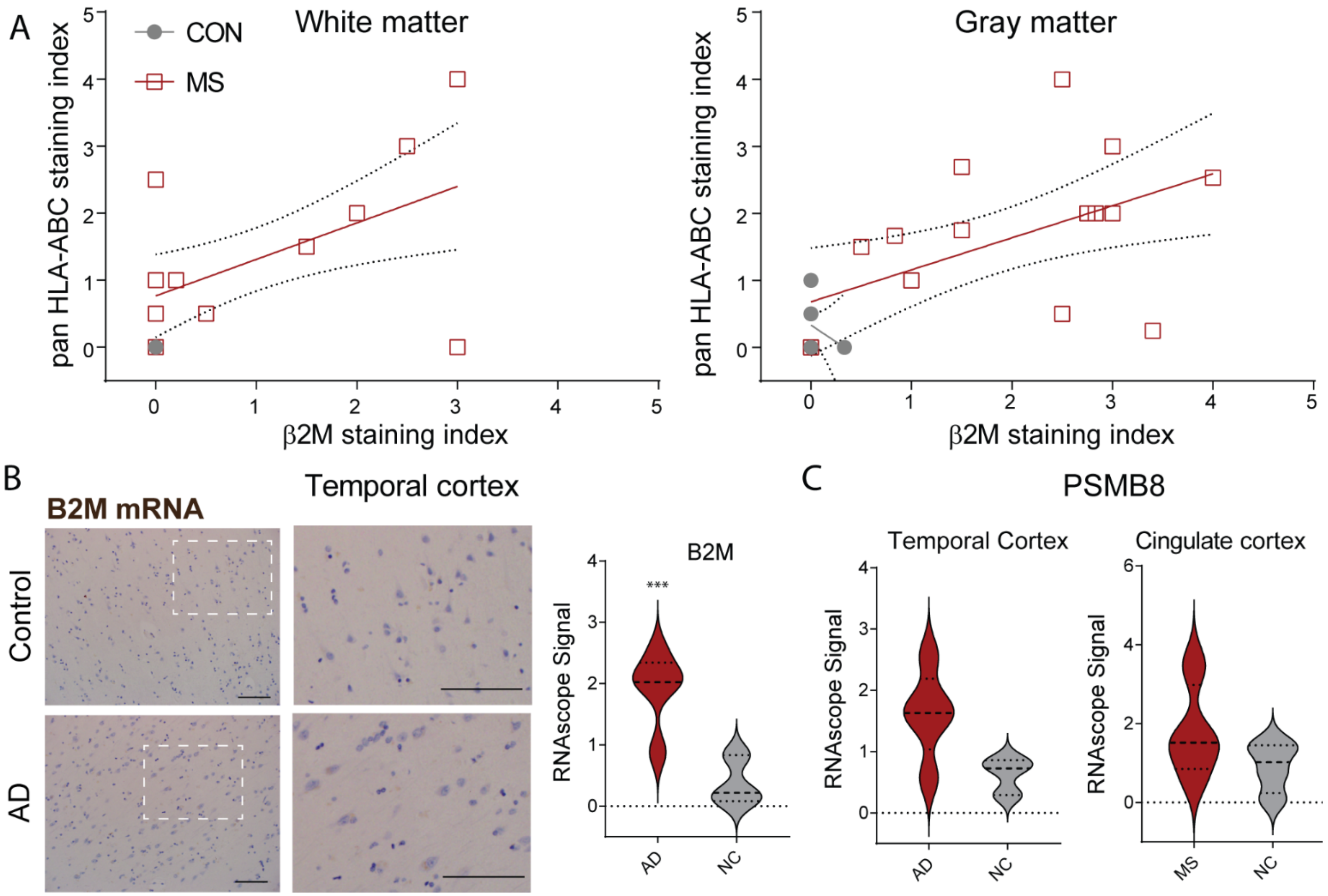

Supplemental Figure 12. **Upregulation of neuronal β2M mRNA in MS and AD brain**. A) Correlation between β2M and HLA-A,B,C staining index across MS patients and controls. Dotted line represents 95% confidence interval. B) Representative micrographs of in situ hybridization for β2M mRNA in temporal cortex tissue from patients with Alzheimer's disease (AD; n = 5) and normal controls (NC; n = 4). Signal quantification shown in violin plots on right. ***P< 0.01. C) Similar quantification of in situ hybridization in paraffin embedded tissue sections from patients with MS (cingulate cortex; n = 4) vs NC (cingulate cortex; n = 3) and AD (temporal cortex; n = 5) vs NC (temportal cortex; n = 4).

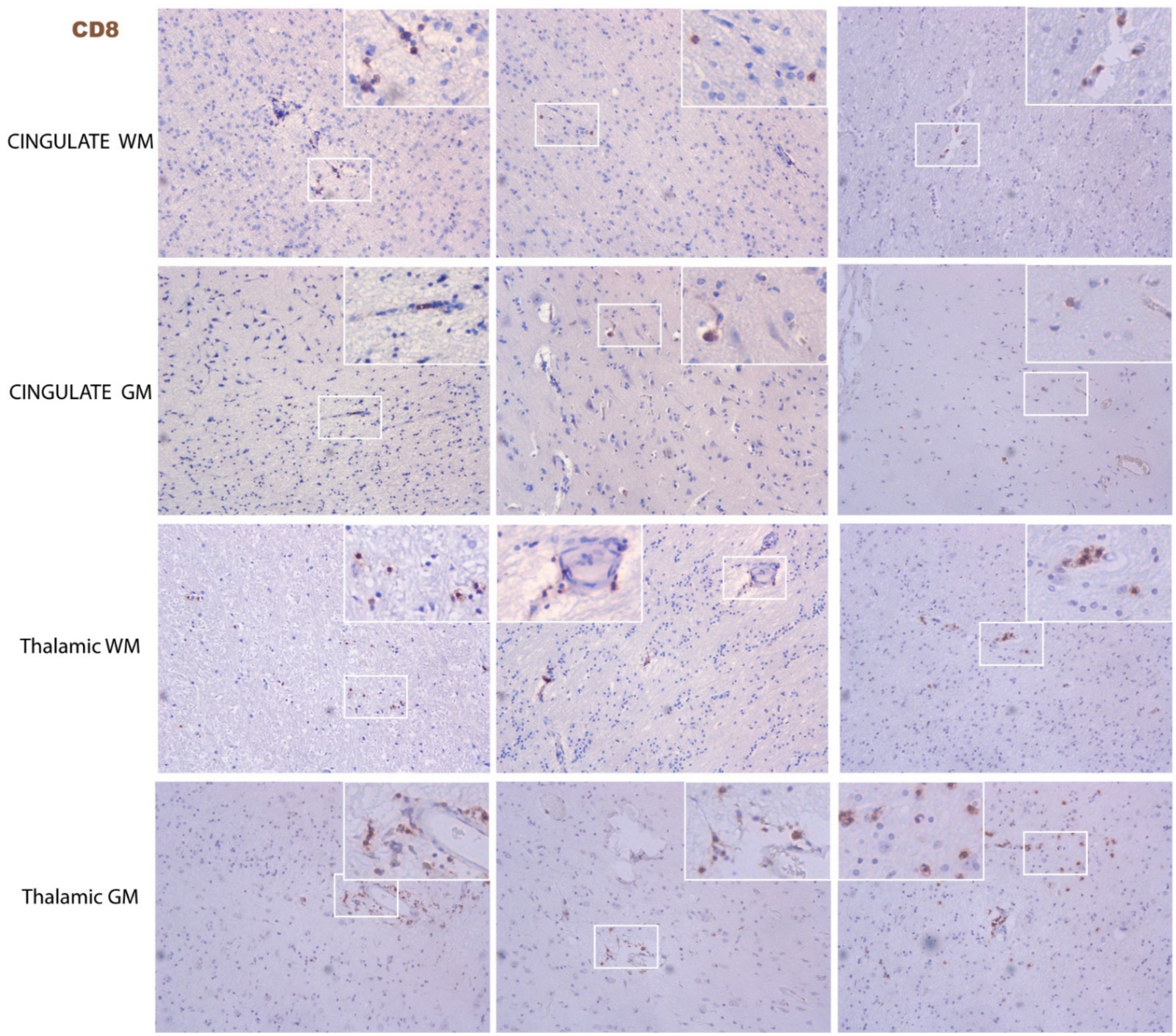

Supplemental Figure 13. Presence of CD8⁺ T cells in normal appearing brain tissues from MS patients. Normal appearing cortical gray matter (cingulate gyrus) and deep gray matter (thalamic nuclei) were immunolabled for CD8. Representative micrographs show presence of $CD8^+$ T cells (brown) in perivascular and parenchymal compartments in these tissues. Prominent infiltration in associated normal appearing white matter was also observed.

Supplemental Table 1: Gene Ontology terms upregulated in neurons by axonal IFNγ stimulation

BP GO Term	Description	Count	P-Value	Fold Enrichment
GO:0002428	Antigen processing and presentation of peptide antigen via MHC class Ib	4	2.00E-06	123.5
GO:0002477	Antigen processing and presentation of exogenous peptide antigen via MHC class Ib	3	1.90E-04	123.5
GO:0002481	Antigen processing and presentation of exogenous protein antigen via MHC class Ib, TAP-dependent	3	1.90E-04	123.5
GO:0002755	MyD88-dependent toll-like receptor signaling pathway	2	1.60E-02	123.5
GO:0002476	Antigen processing and presentation of endogenous peptide antigen via MHC class Ib	2	1.60E-02	123.5
GO:0002483	Antigen processing and presentation of endogenous peptide antigen	3	3.79E-04	92.6
GO:0019883	Antigen processing and presentation of endogenous antigen	3	3.79E-04	92.6
GO:0001916	Positive regulation of T cell mediated cytotoxicity	6	3.72E-09	82.4

Supplemental Table 2: Gene Ontology terms upregulated in demyelinated Syn.Cre-RPL22 mouse cortex

EAE vs CON				
**GO Term**	**Description**	**P-value**	**FDR q-value**	**Enrichment**
GO:0034341	response to interferon-gamma	4.64E-14	6.55E-10	20.7
GO:0002376	immune system process	1.45E-11	1.02E-07	3.33
GO:0034097	response to cytokine	5.98E-11	2.81E-07	6.8
GO:0019882	antigen processing and presentation	1.31E-10	4.60E-07	13.07
GO:0048002	antigen processing and presentation of peptide antigen	1.38E-10	3.90E-07	18.48
GO:0006955	immune response	6.74E-10	1.58E-06	4.06
GO:0035456	response to interferon-beta	9.72E-10	1.96E-06	24.35
GO:0006952	defense response	4.06E-09	7.15E-06	3.51
GO:0002478	antigen processing and presentation of exogenous peptide antigen	5.36E-09	8.40E-06	26.83
GO:0002252	immune effector process	7.73E-09	1.09E-05	5.4
GO:0051707	response to other organism	8.07E-09	1.03E-05	4.8
GO:0071346	cellular response to interferon-gamma	1.27E-08	1.49E-05	18
GO:0098542	defense response to other organism	1.56E-08	1.69E-05	5.16
GO:0071345	cellular response to cytokine stimulus	2.15E-08	2.16E-05	7.52
GO:0019884	antigen processing and presentation of exogenous antigen	2.45E-08	2.31E-05	21.95
GO:0043207	response to external biotic stimulus	6.63E-08	5.84E-05	3.73
GO:0009607	response to biotic stimulus	1.39E-07	1.15E-04	3.57
GO:0002504	antigen processing and presentation of peptide or polysaccharide antigen via MHC class II	1.50E-07	1.17E-04	36.96
GO:0002495	antigen processing and presentation of peptide antigen via MHC class II	1.50E-07	1.11E-04	36.96
GO:0019886	antigen processing and presentation of exogenous peptide antigen via MHC class II	1.50E-07	1.06E-04	36.96
GO:0035458	cellular response to interferon-beta	2.39E-07	1.60E-04	22.18
GO:0022900	electron transport chain	1.10E-06	7.08E-04	12.94
GO:0016064	immunoglobulin mediated immune response	1.85E-06	1.13E-03	23.52
GO:0050896	response to stimulus	2.03E-06	1.19E-03	1.69
GO:0051704	multi-organism process	2.91E-06	1.64E-03	3.2
GO:0006950	response to stress	3.32E-06	1.80E-03	2.04
GO:0045087	innate immune response	3.88E-06	2.03E-03	4.15
GO:0019724	B cell mediated immunity	4.48E-06	2.25E-03	19.9
GO:0009617	response to bacterium	5.01E-06	2.44E-03	5.07
GO:0042832	defense response to protozoan	5.45E-06	2.56E-03	19.17
GO:0022904	respiratory electron transport chain	8.44E-06	3.84E-03	12.42
GO:0002682	regulation of immune system process	1.05E-05	4.64E-03	2.51

GO:0042742	defense response to bacterium	1.07E-05	4.59E-03	5.15
GO:0001562	response to protozoan	1.11E-05	4.61E-03	16.69
GO:0042221	response to chemical	1.58E-05	6.38E-03	2
GO:0002449	lymphocyte mediated immunity	1.64E-05	6.44E-03	11.09
GO:0010033	response to organic substance	2.37E-05	9.03E-03	2.12
GO:0009615	response to virus	3.06E-05	1.14E-02	5.71
GO:0051607	defense response to virus	3.15E-05	1.14E-02	6.57
GO:0002460	adaptive immune response based on somatic recombination of immune receptors built from immunoglobulin superfamily domains	3.56E-05	1.26E-02	9.7
GO:0034340	response to type I interferon	4.81E-05	1.65E-02	38.81
GO:0002819	regulation of adaptive immune response	4.89E-05	1.64E-02	6.18
GO:0002474	antigen processing and presentation of peptide antigen via MHC class I	5.11E-05	1.67E-02	12.32
GO:0009605	response to external stimulus	6.12E-05	1.96E-02	2.41
GO:0050830	defense response to Gram-positive bacterium	6.96E-05	2.18E-02	8.62
GO:0050776	regulation of immune response	7.32E-05	2.24E-02	3.08
GO:0002443	leukocyte mediated immunity	7.52E-05	2.26E-02	8.51
GO:0001914	regulation of T cell mediated cytotoxicity	9.14E-05	2.68E-02	16.56
GO:0002684	positive regulation of immune system process	9.33E-05	2.68E-02	2.78
GO:0048525	negative regulation of viral process	1.26E-04	3.54E-02	7.76
GO:0006120	mitochondrial electron transport, NADH to ubiquinone	1.39E-04	3.83E-02	28.23
GO:0006959	humoral immune response	1.68E-04	4.56E-02	6.04
GO:0002821	positive regulation of adaptive immune response	2.40E-04	6.39E-02	6.9
GO:0001910	regulation of leukocyte mediated cytotoxicity	2.85E-04	7.43E-02	8.62
GO:0002483	antigen processing and presentation of endogenous peptide antigen	2.99E-04	7.67E-02	22.18
GO:0071310	cellular response to organic substance	3.60E-04	9.06E-02	2.29
GO:0045069	regulation of viral genome replication	4.45E-04	1.10E-01	7.84
GO:0019883	antigen processing and presentation of endogenous antigen	4.54E-04	1.10E-01	19.4
GO:0006091	generation of precursor metabolites and energy	5.01E-04	1.20E-01	4.4
GO:0050792	regulation of viral process	5.11E-04	1.20E-01	5.03
GO:0050778	positive regulation of immune response	5.60E-04	1.29E-01	3.3
GO:0055114	oxidation-reduction process	5.62E-04	1.28E-01	2.4
GO:0031341	regulation of cell killing	5.84E-04	1.31E-01	7.39
GO:0045071	negative regulation of viral genome replication	5.89E-04	1.30E-01	10.35
GO:0050829	defense response to Gram-negative bacterium	6.48E-04	1.41E-01	10.1
GO:0035455	response to interferon-alpha	6.52E-04	1.39E-01	17.25
GO:1903036	positive regulation of response to wounding	7.05E-04	1.48E-01	4.77
GO:1903901	negative regulation of viral life cycle	8.50E-04	1.76E-01	6.81
GO:0001916	positive regulation of T cell mediated cytotoxicity	8.98E-04	1.83E-01	15.52
GO:0045351	type I interferon biosynthetic process	9.12E-04	1.84E-01	41.4
GO:0046633	alpha-beta T cell proliferation	9.12E-04	1.81E-01	41.4

CUP vs CON				
GO Term	Description	P-value	FDR q-value	Enrichment
GO:0022900	electron transport chain	3.73E-06	5.27E-02	5.52
GO:0006955	immune response	7.69E-06	5.42E-02	1.96
GO:0022904	respiratory electron transport chain	8.71E-06	4.09E-02	5.62
GO:0048002	antigen processing and presentation of peptide antigen	2.49E-05	8.78E-02	5.02
GO:0071281	cellular response to iron ion	9.95E-05	2.80E-01	14.05
GO:0035456	response to interferon-beta	1.64E-04	3.85E-01	5.78
GO:0002376	immune system process	1.65E-04	3.32E-01	1.56
GO:0032103	positive regulation of response to external stimulus	2.40E-04	4.23E-01	2.27
GO:0002478	antigen processing and presentation of exogenous peptide antigen	3.11E-04	4.88E-01	6.24
GO:0002703	regulation of leukocyte mediated immunity	3.20E-04	4.51E-01	2.69
GO:0046916	cellular transition metal ion homeostasis	4.18E-04	5.36E-01	3.95
GO:0006120	mitochondrial electron transport, NADH to ubiquinone	4.30E-04	5.06E-01	10.21
GO:0034341	response to interferon-gamma	4.70E-04	5.10E-01	3.89
GO:0019882	antigen processing and presentation	5.57E-04	5.61E-01	3.25
GO:0097286	iron ion import	6.28E-04	5.90E-01	9.36
GO:0055076	transition metal ion homeostasis	7.92E-04	6.97E-01	3.12
GO:0042590	antigen processing and presentation of exogenous peptide antigen via MHC class I	8.29E-04	6.88E-01	14.05
GO:0042158	lipoprotein biosynthetic process	8.29E-04	6.49E-01	14.05
GO:0051707	response to other organism	8.45E-04	6.27E-01	1.89
GO:0002699	positive regulation of immune effector process	9.53E-04	6.72E-01	2.52
GO:0019884	antigen processing and presentation of exogenous antigen	9.71E-04	6.52E-01	5.11

Supplemental Table 3: Patient samples for IHC and RNAscope

Group	# samples	Tissue Type
**Immunohistochemistry**		
Multiple Sclerosis	18	Cingulate cortex and Thalamus
Non-MS control	10	Cingulate cortex and Thalamus
**RNAscope**		
Alzheimer’s Disease	5	Temporal Cortex
Non-AD Control	4	Temporal Cortex
Multiple Sclerosis	4	Cingulate Cortex
Non-MS control	3	Cingulate Cortex

Supplemental Table 4: RT-PCR Primers

Gene	Forward Primer	Reverse Primer
H2-D1	aatgtctctccgagattgtaaagc	ggctctggatgtcacagga
H2-K1	atacctgaagaacgggaacg	tgatgtcagcagggtagaagc
PSMB8	gcctatggggtgatggacag	gcaggtcactgacatcggaa
PSMB9	cgctctgctgagatgctg	ctccactgccatgatggtt
TAP1	ttcacccgcaacatatggct	atgtgatggaacctgctggg
TAP2	ggtggcctgctctccttc	ccgtacatgtaaaccaggttcc
TAPBP	tcagcgtatccagcactctc	gaggacggtcagcaccac

Supplemental Table 5: Flow cytometry antibodies

Label(s)	Target	Clone(s)	Location	Source	Cat. No.
FITC	CD4	GK1.5	Surface	BD	553046
APC	CD4	RM4.5	Surface	BD	553051
PerCP-Cy5.5	CD8a	53-6.7	Surface	BD	551162
PE	CD8a	53-6.7	Surface	BD	553033
APC	CD8a	53-6.7	Surface	Biolegend	100733
APC	CD11a (LFA-1)	H155-78	Surface	Biolegend	141010
FITC	CD11a (LFA-1)	M17/4	Surface	Thermo Fisher	11-0111-82
APC-eFluor™ 780	CD25	PC61.5	Surface	Thermo Fisher	47-0251-82
APC	CD44	IM7	Surface	BD	561862
PercP-Cy5.5	CD44	IM7	Surface	BD	560570
FITC	CD44	IM7	Surface	Biolegend	103006
Alexafluor 405	CD44	IM7	Surface	Thermo Fisher	RM5726
Alexafluor 647	CD49a	Ha31/8	Surface	BD	562113
PerCP-Cy5.5	CD69	H1.2F3	Surface	Thermo Fisher	45-0691-82
FITC	CD90.1 (Thy1.1)	OX-7	Surface	BD	561973
APC	CD90.1 (Thy1.1)	OX-7	Surface	BD	561409
FITC	CD103	M290	Surface	BD	557494
FITC	CD107a	1D4B	Surface	BD	561069
Alexafluor 488	CD127 (IL7R)	SB/199	Surface	BD	561533
FITC	Granzyme B	GB11	Intracellular	BD	561998
APC	IFNg	XMG1.2	Intracellular	BD	562018
Alexafluor 647	Ki67	B56	Surface	BD	561126
Alexafluor 488	KLRG1	2F1	Surface	BD	561619
PE	Va2	B20.1	Surface	BD	553289
FITC	Va2	B20.1	Surface	Thermo Fisher	11-5812-82
eFluor™ 450	Vb5.1	MIR9-4	Surface	Thermo Fisher	48-5796-82
APC	Vb5.1	MIR9-4	Surface	Thermo Fisher	17-5796-80

Supplemental Table 6: CyTOF Mouse T-cell Panel

Label	Target	Clone	Location
089Y	CD45	30-F11	Surface
141Pr	CD39	24DMS1	Surface
142Nd	Eomes	Dan11mag	Intracellular/Nuclear
143Nd	TCRb	H57-597	Surface
144Nd	Tcf1	812145	Intracellular/Nuclear
145Nd	CD69	H1.2F3	Surface
146Nd	Gata3	TWAJ	Intracellular/Nuclear
148Nd	ROR gamma (t)	B2D	Intracellular/Nuclear
149Sm	CD366 (Tim-3)	RMT3-23	Surface
150Nd	IRF4	IRF4.3E4	Intracellular/Nuclear
151Eu	CD25 (IL-2R)	3C7	Surface
152Sm	CD3e	145-2C11	Surface
153Eu	CD28	37.51	Surface
154Sm	BATF	D7C5	Intracellular/Nuclear
155Gd	Tbet	4B10	Intracellular/Nuclear
156Gd	CD90.2/Thy-1.2	30-H12	Surface
158Gd	FoxP3	FJK-16s	Intracellular/Nuclear
159Tb	CD279 (PD-1)	RMP1-30	Surface
160Gd	CD62L	MEL14	Surface
161Dy	Ki-67	B56	Intracellular/Nuclear
163Dy	CD4	RM4-5	Surface
164Dy	CD73	TY/11.8	Surface
165Ho	Thy1.1	OX-7	Surface
166Er	CD19	6D5	Surface
167Er	CD38	90	Surface
168Er	CD8a	53-6.7	Surface
169Tm	CD272 (BTLA)	6F7	Surface
170Er	CD161 (NK1.1)	PK136	Surface
171Yb	CD11b		Surface
172Yb	Fas	SA367H8	Surface
173Yb	Bim	K.912.7	Intracellular/Nuclear
174Yb	CD223 (LAG-3)	C9B7W	Surface
175Lu	iNos (NOS2)	CXNFT	Intracellular/Nuclear
176Yb	CD44	IM7	Surface
209Bi	CD11c	N418	Surface

Supplemental Methods

Mice

C57BL/6 wild-type (WT, stock# 000664), B6.PL-Thy1a/CyJ (Thy1.1, stock# 000406), B6.Cg-Tg(Syn1-cre)671Jxm/J (Syn.Cre, stock# 003966), B6.129(Cg)-Gt(ROSA)26Sortm4(ACTB-tdTomato,-EGFP)Luo/J (mT/mG, stock #007676), B6J.129(Cg)-Rpl22tm1.1Psam/SjJ (Rpl22, stock #029977), and C57BL/6-Tg(TcraTcrb)1100Mjb/J (OT-I, stock # 003831) T cell receptor-transgenic mice with SIINFEKL:H2-K^b-restricted $CD8^+$ T cells were obtained from the Jackson Laboratory (Bar Harbor, ME). OT-I mice were crossed with homozygous Thy1.$1^{+/+}$ or mT/m$G^{+/+}$ mice to generate OT-I.Thy1.$1^{+/-}$ or OT-I.mT/m$G^{+/-}$ mice used for adoptive T cell transfer experiments. Rpl22 mice were crossed with Syn.Cre mice to generate neuron-specific hemagglutinin-tagged polyribosomal RNA for microarray analysis. All F1 offspring used in experiments were screened for TCR(Vα2Vβ5) and RFP or Thy1.1 transgene expression by flow cytometry on immune cells isolated from blood. Male and female mice between 6 and 10 weeks of age at the start of experiments were used for all mouse studies.

Cuprizone, EAE, and pertussis toxin injections

Experimental autoimmune encephalomyelitis (EAE) was induced as previously described [2,4,6]. Briefly, an emulsion of equal volumes of complete Freund's adjuvant (CFA) and 100 µg myelin oligodendrocyte glycoprotein peptide ($MOG_{35–55}$, MEVGWYRSPFSRVVHLYRNGK) supplemented with *M. tuberculosis* H37Ra (5 mg/ml) was injected subcutaneously in the scapular region of each mouse (Hooke Laboratories EAE induction kit EK-2110). The MOG-CFA mixture was emulsified by sonication using a sonic dismembrator (Fisherbrand; FB705). Pertussis toxin (200 ng/mouse, i.p.; Hooke Laboratories) was injected on day 0 and 2 relative to immunization. Clinical scores were monitored daily in a blinded manner and recorded as follows: 0, no clinical disease; 1, flaccid tail; 2, gait disturbance or hind limb weakness; 3, hind limb paralysis and no weight bearing on hind limbs; 4, hind limb paralysis with forelimb paresis and reduced ability to move around the cage; and 5, moribund or dead. Only animals exhibiting clinical disease were examined by transcriptional analysis. For experiments modeling blood brain barrier disruption, 200 ng pertussis toxin was injected i.p. in 500 µL PBS as above at 96 hours and 48 hours prior to adoptive transfer of $CD8^+$ T cells. Cuprizone (Bis(cyclohexanone)oxaldihydrazone; Sigma) diet containing 0.3% w/w cuprizone was prepared by Test Diet in 5LG6 base diet and utilized within 6 months of manufacture. Additionally, for initial RiboTag experiments, cuprizone was provided in the diet containing 62.5 ppm encapsulated rapamycin API (Rapamycin Holdings, Inc.) to reduce inflammation. For experiments, mice were allowed access to experimental or control (5LG6) diet ad libitum and monitored weekly for 6 weeks prior to further manipulation. For intracerebral injections, anesthetized mice were injected with 1.5 µL containing $3x10^9$ gene copies of AAV1.Syn.OVA-eGFP or AAV1.Syn.eGFP control 1.0 mm left of bregma at a depth of 1.25 mm below the surface of the brain using a Quintessential Stereotactic Injector pump (Stoelting #53311) at a rate of 0.5 µL/min and a 600 series 5 µL Hamilton Syringe (No. 7633-01). The needle was held in place for 5 min following injection to prevent reflux upon needle removal.

RiboTag translational profiling

For translational profiling, experimental SynCre.Rpl22 mice were perfused transcardially with cold PBS containing 20 µg/mL cycloheximide and 0.1 mg/mL heparin and subsequently processed as previously described to isolate neuronal ribosome-bound mRNA[7]. Briefly, mouse cortex was grossly dissected and homogenized with sterile RNase-Zap treated Dounce homogenizers in 10% w/v homogenization buffer containing 45 mM Tris, 100 mM KCl, 12 mM MgCl2, 1% NP-40, 20 µg/mL cycloheximide, 1 mg/mL heparin, 0.5 % RNasin, 1% protease inhibitor cocktail (Sigma), and 1 mM DTT. Homogenate was centrifuged for 20 minutes at 5000 x g and the supernatant was aliquoted for storage at -80°C. To capture polyribosomes, 7.5 µg/mL HA1.1 monoclonal antibody (16B12; BioLegend 901501) was added to thawed homogenate and incubated with gentle agitation for 4 hours at 4°C. Subsequently, 0.25 volume of Dynal Protein G beads was resuspended in homogenization buffer and added to homogenate for overnight incubation. The next day samples were placed on an EasySep magnet (StemCell Technologies) for 3-5 minutes and the negative fraction was removed. Beads were washed 3 times with high salt buffer containing 45 mM Tris, 300 mM KCl, 12 mM MgCl2, 1% NP-40, 20 µg/mL cycloheximide, and 1mM DTT. Lastly, mRNA was eluted off the bead-bound ribosomes by resuspending the beads in Buffer RLT Plus (Qiagen) and vortexing vigorously. RNA was then isolated using RNeasy Micro Plus spin column assemblies following manufacturer's instructions (Qiagen).

T cell transfers and labeling

For adoptive transfer, $CD8^+$ T cells were purified from lymph nodes of B6 or OT-I mice. Lymph nodes were gently dissociated, and cells passed through a 40-micron cell strainer prior to magnetic isolation of $CD8^+$ T cells (Miltenyi 130-095-236). Briefly, single cell suspensions were counted, brought up to 2 x 10^7 cells per mL, labeled with antibody cocktail containing biotinylated anti-CD11b (M1/70), anti-B220 (RA3-6B2), and anti-CD4 (GK1.5) at 4 µg/mL each for 30 minutes at room temperature, washed, and resuspended in 50 □L/10^7 cells of solution containing Streptavidin Plus microparticles (BD). After 15 minutes cells were diluted and placed on an EasySep magnet for 6 minutes. Eluates were placed on the magnet for another 4 minutes, eluted once again, counted, and resuspended in 50% FBS/ PBS at 10^7 cells per mL for i.v. injection. Recipient mice were irradiated with 400 rads 4 hours prior to i.v. injection of 200 µL containing 2 x 10^6 $CD8^+$ T cells. $CD8^+$ T cell purity was confirmed by immunostaining and flow cytometry. For experiments involving transfer of labeled cells, $CD8^+$ T cells were labeled with 5 µM carboxyfluorescein succinimidyl ester (CFSE) or 5 µM 5-chloromethylfluorescein diacetate (CMFDA; Cell Tracker Green, ThermoFisher Scientific) in RPMI 1640 containing 1% HEPES for 20 minutes at 37°C and then quenched with 10 volumes heat inactivated FBS. Cells were washed and resuspending in 50% FBS/PBS for i.v. injections as above. Recipient mice were not irradiated for these experiments to prevent homeostatic expansion of transferred cells.

Patient Samples
Paraffin-embedded postmortem brain tissue sections were obtained from the Mayo Clinic Tissue Registry, Mayo Clinic Alzheimer's Disease Research Center, Normal Aging Brain Collection Amsterdam, and the Netherlands Brain Bank (Supplemental Table 3). For IHC, we analyzed samples from controls (n = 6 females and n = 4 males) aged 66 ± 6 (mean ± SD) years of age and samples from secondary progressive MS patients (n = 16 females and n = 2 males) aged 66 ± 7 years of age. Immediately prior to immunostaining or in situ hybridization 10-micron thick sections were prepared by the Mayo Clinic Pathology Research Core facility.

Cortical neuron cultures and microfluidic devices
Tissue-culture treated vessels were coated with 0.5 mg/mL poly-ornithine in 100 mM borate buffer. Cortical neurons were prepared from C57BL6 mouse embryonic day 15 (E15) pups as previously described [2,9]. Briefly, the upper halves of the cerebral cortices were dissected, and meninges were removed and washed in Hank's balanced salt solution (HBSS). The tissue was washed in neuron plating media, which contained high-glucose Dulbecco's modified Eagle medium (DMEM) with glutamine, supplemented with 10% bovine calf serum and 10% F12 supplement. Cortices were digested in 2 mg/mL papain in HBSS at 37°C for 15 minutes, serially triturated with a 5 mL serological pipet, a P1000 pipet tip, a glass Pasteur pipet, and finally a fire polished glass Pasteur pipet, until the tissue was roughly dissociated into a single cell suspension. Cells were centrifuged at 400 x g for 4 min and seeded at 4.5 x 10^5 cells per cm^2 (10^6 cells per mL) on poly-ornithine coated plates. In some experiments, cells were infected at plating with 2000 MOI (2 x 10^9 gc/mL) of various adeno-associated viruses (AAVs). Three hours after seeding, the cortical neurons were fed with 2 volumes of neuron feed media containing neurobasal media supplemented with 2% B27, 1% Glutamax, and 100 U/mL penicillin and streptomycin. During the first 48 hours cells were given 1 ng/mL brain derived neurotrophic factor (BDNF) and 10 ng/mL insulin-like growth factor 1 (IGF1). Subsequently, neurons were maintained in neuron feed media by changing half of the media volume every 2-3 days. In some experiments, neurons were cultured in polydimethylsiloxane (PDMS) microfluidic axon isolation chambers, as previously described [9]. Microfluidic chambers were fabricated by pouring PDMS (Sylgard184 Elastomer, Dow Inc.) plus 10% v/v crosslinking reagent onto silicon wafer molds etched by soft lithography (SimTech Microfluidics foundry). The dimensions of these chambers are described elsewhere [10]. Neuronal progenitor cells (2 x 10^5) were plated in the cell body chambers in plate media and fed 3 hours later with neuron feed media containing BDNF and IGF1, as above. Neuron feed media without BDNF and IGF was replaced every 2-3 days in the cell body chamber and media containing BDNF and IGF1 was added every 2-3 days to the distal chamber to support trophic factor-driven directional outgrowth of axons into the distal chamber.

Immunohistochemistry and in situ hybridization
Tissue sections (10 microns thick) were stained with hematoxylin and eosin (H&E) or immunostained as follows. After deparaffinization and rehydration, sections were treated with 3% hydrogen peroxide in Tris-buffered saline (TBS) for 30 minutes and then exposed to heat-induced antigen retrieval in Tris-EDTA (10 mM/1 mM; pH 9.0) or citrate buffer (10 mM pH 6.0) for 30 minutes at 95°C. Sections were blocked for 30 min with 3% bovine serum albumin and 2.5% serum from the secondary antibody host species in TBS and then stained with primary antibodies overnight at 4°C. For primary analysis, 5 µg/mL rabbit anti-β2M (13511-1-AP

Invitrogen) or 1:200 mouse anti-pan-HLA-A,B,C (W6/32; Ebiosciences 14-9983-82) antibodies were used. Inflammation was evaluated on serial H&E sections and by staining with 1:400 mouse anti-HLA-DR (LN3; ThermoFisher Ma5-11966) antibody. Demyelination was assessed by staining with 1:500 mouse anti-PLP (PLPc1; Biorad MCA839G) antibody. Protein adsorption was used as a negative control during validation. After several washes, secondary antibodies (biotin-SP donkey anti–rabbit or biotin-SP donkey anti–mouse) were applied to sections and incubated for 2 hours at room temperature. Staining was developed using the VECTASTAIN® Elite® ABC HRP Kit (Vector Laboratories) with 0.5 mg/mL diaminobenzidine (Sigma) and 0.01% H_2O_2. Slides were lightly counterstained with hematoxylin, rinsed with running tap water, and mounted with toluene. Automated in situ hybridization was performed by the Mayo Clinic Pathology Research Core following optimization and validation using the following RNAscope 2.5 LS Probes (ACD Bioteche) with RNAscope 2.5 Reagent Kit Brown (#322100) in accordance with the manufacturer's instructions: Hs-β2M (#310168), Hs-TAP2 (#494308), and Hs-PSBM8 (#546838). Hs-PPIB (#313908) and dap-B (#312038) probes were used as positive and negative probes on all tissues to confirm RNA integrity and sample quality. Images were acquired on a CKX41 microscope equipped with a CP73 camera (Olympus) using Cell View image acquisition software. Micrographs with excessive background or tissue folding were excluded from subsequent analyses. For semiquantitative analysis of in situ hybridization, digital images were processed and analyzed using Fiji ImageJ software as previously described [8]. Briefly, following image deconvolution and DAB channel thresholding, mean staining intensity was measured. Average values (representative of 5-7 micrographs) are reported for each patient. All contrast manipulations were applied equally to each image.

Western blot analysis
For Western blot analysis mouse cortical neuron cultures were infected at plating with AAV vectors. Protein lysates were prepared from these cultures at DIV 12-14 using lysis buffer containing Nonidet P40 (1% v/v), deoxycholic acid sodium salt (0.5% w/v), and glycerol (10% v/v) plus the protease inhibitors NaF (1 mM), $NaVO_3$ (1 mM), Aprotinin (10 µg/mL), Leupeptin (1 µg/mL), and phenylmethylsulfonyl fluoride (1 mM). Lysates were denatured for 5 minutes at 95 C in laemmli buffer (1:1) and 30 uL loaded into each well of a 4-20% criterion gel. After electrophoresis, proteins were transferred to polyvinylidene difluoride membrane, blocked for 1 hour with 5% bovine serum albumin in TBS, and probed with primary mouse anti-ovalbumin antibody (3G2E1D9,3D5F4A8,3G1E1D9, 1:2000) in block buffer overnight at room temperature. Afterward blots were washed and probed with secondary antibody (horse radish peroxidase conjugated donkey anti-mouse; 1:5000 Jackson Immunolab) in block buffer for 60 minutes at room temperature. After extensive washing blots were developed with Supersignal West Pico Plus Chemiluminescent Substrate and acquired on a Chemidoc (BioRad) by luminescence imaging or CL-XPosure™ Film on X-OMAT (Kodak). Blots were stripped with pH 1.85 TBS for 10-15 minutes for reprobing with anti-beta actin primary (1:5000) for loading control following the protocol above.

RTPCR, microarray, and gene set enrichment analysis
Cortical neuron lysates were dissociated and homogenized using QIAShredder (Qiagen), following the manufacturer's instructions. RNA was isolated using RNeasy micro plus kit (Qiagen) and genomic DNA was excluded using gDNA eliminator spin column. Expression of the indicated genes was quantitated relative to the house keeping gene *UROD* or *GAPDH*. Gene specific primers (Supplemental Table 4) were selected using NCBI Primer Blast and 20 µL reactions using SsoAdvanced Universal SYBR Green Supermix (BioRad) were carried out following the manufacturer's instructions. Microarray analysis of neuronal transcripts following axonal treatment with IFNg in microisolation chambers was performed at the Mayo Clinic Gene Expression core facility using MouseWG-6 v2.0 Gene Expression BeadChip according to the manufacturer's instructions. Raw data was log2 transformed and analyzed in R with Significance Analysis of Microarrays (SAM; Standford University) using an FDR < 5%. Ribotag microarray analysis was performed at the University of Illinois at Chicago Core Genomics Facility. Briefly, RNA was isolated from neuronal ribosomes as described above and quality control was performed using an Agilent TapeStation system. Total RNA was then used to create the biotin-labeled library to be hybridized on GeneChip® Mouse Gene 2.0 ST Arrays (Affymetrix) microarrays containing the entire mouse transcriptome (34 K transcripts) including ~ 26500 coding and >3500 non-coding transcripts. Following the manufacturer's protocols a total of 16 microarray chips were used for 6 EAE, 6 Cuprizone and 4 control samples with quality control verification before amplification and before hybridization. The arrays were finally scanned in an Affymetrix GeneTitan System. Intensities of target hybridization to respective probe features were detected by laser scanning of the array. Raw data (CEL files) were imported into Transcriptome Analysis Console (Affymetrix) and normalized by robust multiarray average (RMA).

Statistical analysis was performed considering an FDR <20%. Gene ontology analysis of microarray data (ranked gene lists) was performed in Gene Ontology enrichment analysis and visualization software (GORILLA). Only biological process terms with fewer than 50 genes are reported along with p values and fold enrichment.

Tissue processing for immunofluorescence and confocal imaging
For immunostaining, mice were deeply anesthetized and then perfused transcardially with > 15 mL heparinized saline solution followed by > 30 mL 4% paraformaldehyde (PFA) solution. Brains and cervical lymph nodes were collected and further post fixed in 4% PFA for 24 hours. Brains were then washed and transferred to 30% sucrose solution prior to embedding. For floating sections, brains were embedded in 4% low melting point agarose in PBS and 70-micron floating sections were cut on VT1000 P Manual vibratome (Leica). Floating sections were permeabilized overnight in 0.1% Triton-X 100 in TBS and then transferred to blocking buffer containing 2.5% normal donkey serum, 5% bovine serum albumin, and 0.1% Triton-X 100 in TBS for 2 hours. Floating sections were then transferred to blocking buffer containing primary antibodies (5 ug/mL), washed again and counterstained with DAPI prior to mounting with VECTASHIELD Antifade Mounting Media (Vector Biolabs). For cryosections (8-20 micron), tissues were instead embedded in cryomolds with optimal cutting temperature (OCT) compound. Sections were cut at -18C to -20C on a cryostat, transferred to subbed slides and immunostained as above. For CD8 staining, sections were not permeabilized and Triton-X 100 was excluded from staining and wash buffers.

**Antibodies used for mouse tissues**			
**Target**	**Clone(s)**	**Source**	**Cat. No**
HA.11 Tag	16B12	Biolegend	901501
H2Db/kb	28-8-6	BD Bioscences	553576
H2Db	28-14-8	BD Bioscences	553600
H2Kb	AF6-88.5	BD Bioscences	553567
H2Kb:SIINFEKL	25-D1.16	Ebiosciences	130-096-810
Beta Actin	pAb	Cell Signaling Technologies	4967
CD11c	HL3	BD Bioscences	550283
Lyve1	pAB	Novus Biologicals	Nb100-725SS
CD8a	5H10	Biolegend	100802
CD45	30F11	BD Bioscences	550539
CD3	17A2	BD Bioscences	555273
APP	22C11	Millipore Sigma	MAB348
npNF	SMI32	Covance; now Biolegend	SMI32p

Tissue processing for flow cytometry and intracellular cytokine staining
For flow cytometric analysis, leukocytes were isolated from brain, blood, deep cervical lymph nodes, or spleen as previously described[4-6]. Briefly, blood (100 µL) was collected transcardially with an insulin syringe prior to perfusion with > 15 mL heparinized saline solution. Blood was transferred to a 15 mL conical tube containing 10 mL Hanks balanced salt solution with 10 mM EDTA and spun at 400g for 5 minutes. Pellets were resuspending in HBSS, underlaid with 2 mL Histopaque-1077 and centrifuged at 600 g for 20 minutes to remove red blood cells. White blood cells were collected from the interface and upper layer, washed, and used for downstream analysis. Following, perfusion, lymph nodes and spleen were gently dissociated in RPMI 1640 and passed through 40-micron cell strainers. Splenocytes were washed and resuspending in 1mL ACK containing RBC lysis buffer for 1 minute and then washed again with > 10 ML RPMI 1640 prior to counting. For immunostaining 10^6 lymphocytes or splenocytes were used from each mouse for each staining panel. Brains were minced with straight razors in RPMI 1640 and then gently dissociated in tissue homogenizers and spun at 400g for 5 minutes. Pellets were resuspending in 70% Percoll 30% RPMI and overlaid with 30% Percoll 70% PBS prior to centrifugation at 2200 g for 30 minutes with no brake. Leukocytes were collected from the interface, washed with > 10 volumes RPMI and split evenly across staining panels or ex vivo assays. For surface staining, single cell suspensions were washed with FACS buffer containing 1% bovine serum albumin, 2 mM EDTA in PBS and then incubated for 30 minutes on ice in FACS buffer containing Fc Block (BD) or 25% v/v 2.4G2 cell supernatant and saturating concentrations of fluorophore conjugated monoclonal antibodies

(Supplemental Table 5). Cells were washed three times with FACS buffer and acquired on an Attune NxT (ThermoFisher) or C6-Accuri (BD) flow cytometer equipped with a 488 nm laser (filter set: 533/30, 585/40, 670LP) and a 640 nm laser (filter set: 675/25, 780/60). For intracellular cytokine staining, following surface staining cells were fixed and permeabilized overnight at 4C in BD Fix/Perm buffer, washed with 1X Perm Wash solution and then stained with 5 ug/ml anti-IFNγ APC in 1X Perm Wash solution. Cells were washed three times with 1X Perm Wash solution prior to acquisition.

Ex vivo assays
For ex vivo assays, leukocytes were first isolated from tissues as described above for flow cytometry. Recall assays were performed as previously described[4,5]. Briefly, brain infiltrating leukocytes were washed and cultured for 5 hours in RPMI 1640 media containing 3% FBS and monensin (GolgiStop; 1:1000) plus the indicated treatments (10 ug/mL SIINFEKL or 50 ng/mL phorbol myristic acid and 1 µM ionomycin). Afterward cells were washed and stained for surface markers and then for IFNγ by intracellular cytokine staining as described above. For ex vivo cytokine secretion assays, CD8+ T cells were isolated from brain infiltrating leukocytes by negative selection using magnetic beads (Miltenyi), counted, and plated in 50 µL 3% FBS RPMI 1640 in U-bottom wells of a 96 well plate (1000 cells per well). The next day supernatants were collected for analysis by cytometric bead array as described below. For axon killing assays, mouse cortical neurons were plated in microfluidic axon isolating chambers and infected at 2000 MOI with AAV1.Syn.OVA-eGFP or AAV1.Syn.eGFP empty vector control. Cells were monitored for axon outgrowth and treated with IFNγ (100 ng axon chamber) 72 hours prior to DIV 15, which coincided with tissue collection time points for in vivo experiments. CD8+ T cells were magnetically isolated from brain leukocytes or 100 µL blood from each mouse as above and distributed to 3 wells of a U-bottom 96 well plate. Cells were placed in an incubator and allowed to settle to the bottom of the plate (<30 minutes). Then we collected 10 µL of T cells directly from the bottom of each well and transferred these cells to a unique microfluidic chamber containing AAV1.Syn.OVA-eGFP+ or AAV1.Syn.eGFP+ axon fields. Axon fields were acquired by fluorescent imaging prior to T cell treatment and at 90 minutes, 24 hours, 48 hours, and 72 hours post treatment for analysis. Axon injury index was scored using ImageJ software to quantify the area (sq. mm per field) of axonal blebbing defined by small (30-1000 pixels) rounded (0.3-1.0 circularity) areas separate from intact GFP+ axons.

Cytometric bead array analysis
Cytometric bead array analysis of proinflammatory cytokines was performed on a C6-Accuri flow cytometer (BD) using Mouse Inflammation CBA kit (BD) according to the manufacturer's instructions. Briefly, 50 µL cell supernatants or standards were incubated for 2 hours at room temperature with 50 µL containing each of 6 capture beads against IL10, IL12p70, TNFα, CCL2, IFNγ, and IL6. PE-detection reagent containing PE-labeled antibodies against the cytokines above was then added to these samples (50 µL) for another 2 hours, after which samples were spun down, washed, and acquired. Analysis was performed in FCAP array v3.0 software using 6th order polynomial equations generated from standard curves for interpolation of unknowns.

CyTOF acquisition and analysis
Cytometry by time-of-flight analysis was performed by the Mayo Clinic Immune Monitoring core following magnetic bead based negative selection for CD8+ T cells using CD8+ T cell isolation kit (Miltenyi) on leukocytes isolated from brain and blood as described above. Briefly, $4x10^6$ cells were resuspended in 1 mL of Cell Staining Buffer (CSB; Fluidigm). Each sample was incubated for 5 minutes with 0.5 mm Cisplatin solution in PBS. Samples were then washed twice with CSB. An antibody cocktail (Supplemental Table 6) of the entire phenotyping panel was prepared as a master mix prior to adding 50 mL of cocktail to samples resuspended in 50 mL of CSB. Custom conjugated antibodies were generated in-house through the Mayo Clinic Hybridoma Core using Maxpar X8 Ab labeling kits (Fluidigm) according to the manufacturer's protocol. Samples were then incubated at room temperature for 45 minutes. After washing twice with CSB, samples were fixed with 2% PFA in PBS (Fix and Perm Buffer; Fluidigm). After fixation and wash, samples were resuspended in 30 nM intercalation solution (Cell-ID Intercalator-Ir; Fluidigm). Afterwards 30 mL of unique barcoding reagent (Cell-ID™ 20-Plex Pd Barcoding Kit; Fluidigm) was added to each sample and incubated overnight at 4°C. On the following morning cells were washed with PBS and pooled prior to resuspension in a 1:10 solution of calibration beads (EQ Four Element Calibration Beads; Fluidigm) and Cell Acquisition Solution (CAS; Fluidigm) at a concentration of $0.5x10^6$ cells/mL. Prior to data acquisition samples were filtered through a 35 µm blue cap tube (Falcon). Samples were loaded onto a Helios CyTOF® system (Fluidigm) using an attached autosampler and were acquired at a rate of 200-400 events per second. Data were collected as .FCS files using the Cytof

software (Version 6.7.1014). After acquisition intrafile signal drift was normalized to the acquired calibration bead signal using the Cytof software. Cleanup of cell debris, removal of doublets and dead cells was performed using FloJo software version 10.5.3 (Ashland, OR). Cleaned fcs files were analyzed by the R-based tool Cytofkit version 3.8. Mean signal intensity is shown for the indicated markers on transferred CD45+ CD3+ CD8+ Thy1.1+ T cells.

Statistics
All graphs show mean and 95% confidence intervals unless otherwise indicated. P values less than 0.05 were considered significant. Normality was determined by the Shapiro–Wilk test or Kolmogorov-Smirnov test. For multiple comparisons one-way or two-way analysis of variance (ANOVA) or non-parametric (Kruskal-Wallis) tests were performed where appropriate. Reported P values were corrected for multiple comparisons (Holm-Sidak, Dunnett's, or Benjamini-Hochberg correction for ANOVA; Dunn's correction for Kruskal-Wallis; Benjamini-Hochberg for multiple unpaired t-tests). Unpaired two-tailed Student's t-tests were used for comparisons made between two groups.

Study approval
All animal experiments were approved by the Mayo Clinic institutional animal care and use committee in accordance with National Institutes of Health guidelines. Study approval for human tissues was granted by the Mayo Clinic institutional review board (IRB# 18-008128).

Data and Material Availability
All data are available in the main text or the supplementary materials. Individual data points for each figure are available in the "Supporting Data Values.xls" file. MIAME-compliant microarray data are deposited with the Gene Expression Omnibus under accession number GSE241781. Please contact the author for any further material or data requests.

References

1. Sauer, B.M., Schmalstieg, W.F. & Howe, C.L. Axons are injured by antigen-specific CD8(+) T cells through a MHC class I- and granzyme B-dependent mechanism. *Neurobiol Dis* **59**, 194-205 (2013).
2. Clarkson, B.D.S., Kahoud, R.J., McCarthy, C.B. & Howe, C.L. Inflammatory cytokine-induced changes in neural network activity measured by waveform analysis of high-content calcium imaging in murine cortical neurons. *Sci Rep* **7**, 9037 (2017).
3. Clarkson, B.D.S., Patel, M.S., LaFrance-Corey, R.G. & Howe, C.L. Retrograde interferon-gamma signaling induces major histocompatibility class I expression in human-induced pluripotent stem cell-derived neurons. *Ann Clin Transl Neurol* **5**, 172-185 (2018).
4. Clarkson, B.D.*, et al.* CCR2-dependent dendritic cell accumulation in the central nervous system during early effector experimental autoimmune encephalomyelitis is essential for effector T cell restimulation in situ and disease progression. *J Immunol* **194**, 531-541 (2015).
5. Clarkson, B.D.*, et al.* CCR7 deficient inflammatory Dendritic Cells are retained in the Central Nervous System. *Sci Rep* **7**, 42856 (2017).
6. Clarkson, B.D.*, et al.* Mapping the accumulation of co-infiltrating CNS dendritic cells and encephalitogenic T cells during EAE. *J Neuroimmunology* **277**, 39-49 (2014).
7. Sanz, E.*, et al.* Cell-type-specific isolation of ribosome-associated mRNA from complex tissues. *Proc Natl Acad Sci U S A* **106**, 13939-13944 (2009).
8. Crowe, A.R. & Yue, W. Semi-quantitative Determination of Protein Expression using Immunohistochemistry Staining and Analysis: An Integrated Protocol. *Bio Protoc* **9**(2019).
9. Sauer, B.M., Schmalstieg, W.F. & Howe, C.L. Axons are injured by antigen-specific CD8(+) T cells through a MHC class I- and granzyme B-dependent mechanism. *Neurobiol Dis* **59**, 194-205 (2013).
10. Clarkson, B.D.S., Patel, M.S., LaFrance-Corey, R.G. & Howe, C.L. Retrograde interferon-gamma signaling induces major histocompatibility class I expression in human-induced pluripotent stem cell-derived neurons. *Ann Clin Transl Neurol* **5**, 172-185 (2018).

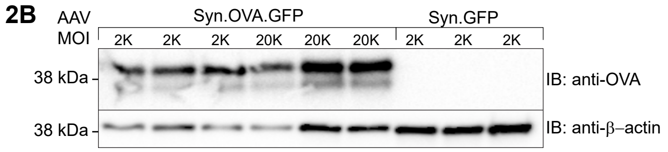

Date: 09/20/2016
Experiment: Ova WB (Ctx neurons, 6-well)
Antibody: Ova (MA5-15307), GFP (N86_38), b-actin
Expected size: </=27 - 45 kDa, 27 kDa, 42 kDA

Sample Prep: NDG+PI's
Gel %: AnykD™ Criterion™ TGX Stain-Free™ Protein Gel, 18 well, 30 µl #5678124
20ul/well = 40ul/sample (prep 50ul)

1	2	3	4	5	6	7	8	9	10	11	12	13	14	15	16	17	18
Ladde r </= 10ul	OVA fl 1:1000 0	Ctx Lysat e	ON Lysat e x6	ON Lysat e x2	2k Ova+7 2 IFN 3	2k Ova+7 2 IFN 2	2k Ova+7 2 IFN 1	2k Ova+2 4 IFN 2	2k Ova+2 4 IFN 1	2k Ov a ctrl	20k Ova+2 4 IFN	20k Ov a 2	20k Ov a 1	eGFP+2 4 IFN	eGFP 2	eGF P 1	Ladde r 12ul

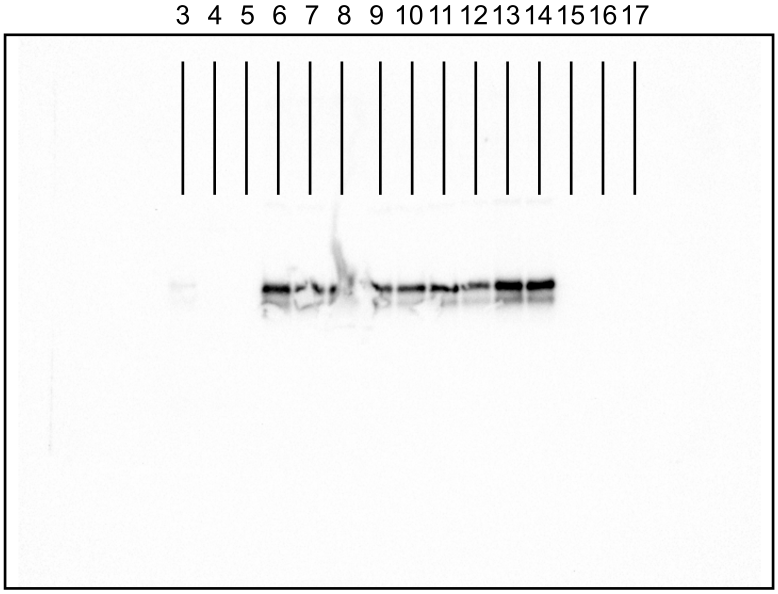

anti-OVA

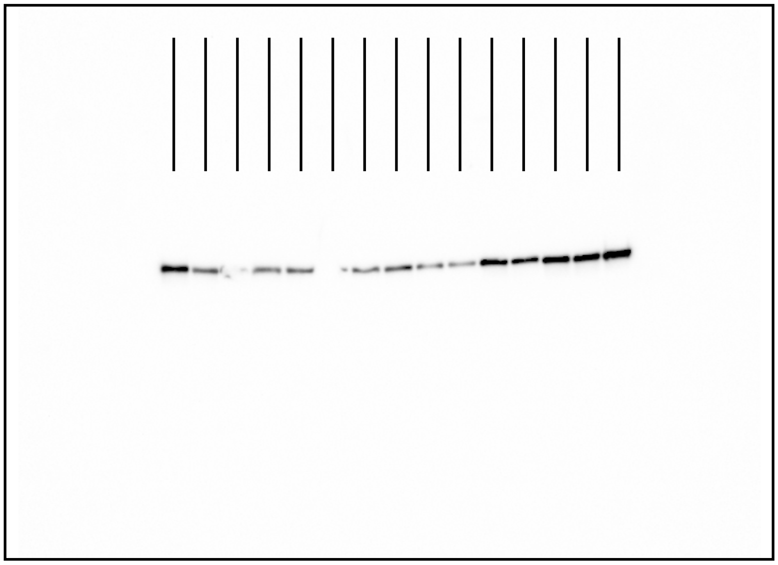

anti-actin
